# A screen for stress-induced sleep genes in *Caenorhabditis elegans* reveals a role for glutamate signaling

**DOI:** 10.1093/genetics/iyag130

**Published:** 2026-05-20

**Authors:** Quinn Howe, Caroline Kominick, Mercedes Pierce, Gina Gazzara, Caroline E Curtin, Santino Diana, Fredy J Abboud, Jordan Olenginski, Mary Frattara, Ting Brown, Teagan McCarthy, Paige Conrad, Lauren Yoslov, Rachel Vemula, Anthony Gargani, Emily Le, Matthew D Nelson

**Affiliations:** Department of Biology, Saint Joseph's University, Philadelphia, PA 19131, United States; Department of Biology, Saint Joseph's University, Philadelphia, PA 19131, United States; Department of Biology, Saint Joseph's University, Philadelphia, PA 19131, United States; Department of Biology, Saint Joseph's University, Philadelphia, PA 19131, United States; Department of Biology, Saint Joseph's University, Philadelphia, PA 19131, United States; Department of Biology, Saint Joseph's University, Philadelphia, PA 19131, United States; Department of Biology, Saint Joseph's University, Philadelphia, PA 19131, United States; Department of Biology, Saint Joseph's University, Philadelphia, PA 19131, United States; Department of Biology, Saint Joseph's University, Philadelphia, PA 19131, United States; Department of Biology, Saint Joseph's University, Philadelphia, PA 19131, United States; Department of Biology, Saint Joseph's University, Philadelphia, PA 19131, United States; Department of Biology, Saint Joseph's University, Philadelphia, PA 19131, United States; Department of Biology, Saint Joseph's University, Philadelphia, PA 19131, United States; Department of Biology, Saint Joseph's University, Philadelphia, PA 19131, United States; Department of Biology, Saint Joseph's University, Philadelphia, PA 19131, United States; Department of Biology, Saint Joseph's University, Philadelphia, PA 19131, United States; Department of Biology, Saint Joseph's University, Philadelphia, PA 19131, United States

**Keywords:** sleep, *C. elegans*, neuropeptides, glutamate

## Abstract

Sleep is an essential behavioral state that evolved early in animals, possibly with the advent of the nervous system. The complexity of sleep neural networks varies significantly across phylogeny, yet common signaling molecules exist. Stress-induced sleep (SIS) of *Caenorhabditis elegans* is controlled by 2 sleep interneurons (ALA and RIS), within a 302-celled nervous system. Even in this simple framework, a complex array of signaling molecules is expressed. Here, we surveyed some of these genes for roles in SIS. These included neuropeptides, G-protein coupled receptors, a 2-pored potassium channel, and glutamate signaling components. We found that multiple genes are required for sleep maintenance (i.e. amounts) and/or the precise timing of sleep initiation. In particular, we identified an important role for glutamate signaling. The conserved ionotropic glutamate receptor *glr-5* regulates sleep maintenance and timing and is required in a 3-celled circuit of interneurons connected by gap junctions and chemical synapses with RIS. This work suggests that numerous redundant and/or parallel mechanisms have evolved to modulate a simple sleep-regulating circuit in *C. elegans*, and we speculate that conserved pathways may play similar roles in animals with more complex systems.

## Introduction

Sleep exists in all animals with nervous systems (Anafi et al. 2019) and may even have evolved in ancestral animals who lacked true tissues, considering parazoans express circadian and synaptic transmission genes (Rivera et al. 2012; Musser et al. 2021). Conserved sleep-regulatory molecules are found even in primitive animals with radially arranged nerve nets, like hydra (Kanaya et al. 2020) and jellyfish (Nath et al. 2017), suggesting that this mysterious behavioral state evolved once, prior to the appearance of the bilaterians. Thus, conserved mechanisms may regulate sleep across phylogeny (Trojanowski and Raizen 2016; Anafi et al. 2019), despite dramatic differences in nervous system complexity. Invertebrate models have emerged as powerful tools for sleep gene discovery and mechanistic dissection.

The nematode *Caenorhabditis elegans* has a 302-cell, fully mapped nervous system (White et al. 1986; Taylor et al. 2021) and displays sleep controlled by conserved genes (Honer and Nelson 2024). Stress-induced sleep (SIS) is a particularly intriguing model for sleep gene identification, considering its experimental reproducibility, and control by just 2 interneurons, ALA (Hill et al. 2014) and RIS (Konietzka et al. 2020). SIS provides restorative functions required for recovery following injury or infection, akin to sickness sleep of mammals (Davis and Raizen 2017). Extreme temperatures, ethanol, pore-forming toxins, hyperosmotic conditions (Hill et al. 2014), wounding (Goetting et al. 2020), exposure to the bacterial toxin indole (Diya et al. 2025), viral infection (Iannacone et al. 2024), and ultraviolet (UV) irradiation (DeBardeleben et al. 2017) can lead to SIS. Noxious stimuli can damage tissues differently, but a common behavioral program occurs: (i) avoidance and escape; (ii) sleep, and (iii) arousal. The timing and amount (i.e. maintenance) of sleep depends on the extent of cellular damage (Hill et al. 2014); thus, temporal dynamics can vary.

Numerous signaling mechanisms must coordinate the opposing states of sleep and arousal. Some of the most critical molecules of sleep regulation are epidermal growth factors (EGF) coded by *siss-1* (Hill et al. 2024), which are shed from damaged tissues to activate EGF receptors, coded by *let-23*, on the surface of the ALA (Van Buskirk and Sternberg 2007), and RIS (Konietzka et al. 2020). Wounding of skin promotes ALA and RIS activation by *siss-1* as well as by the upregulation and release of antimicrobial peptides (Pujol et al. 2008; Sinner et al. 2021). ALA and RIS activation leads to the secretion of a collection of neuropeptides required for sleep behaviors like movement, feeding, and defecation quiescence. These include *nlp-8*, *nlp-14*, *flp-13*, and *flp-24*, expressed in the ALA (Nelson et al. 2014; Nath et al. 2016; Honer et al. 2020), and *flp-11* in the RIS (Turek et al. 2016; Konietzka et al. 2020). Upon secretion, these peptides may function to inhibit wake and motor interneurons (Iannacone et al. 2017; Cianciulli et al. 2019) and cholinergic motor neurons (Rossi et al. 2025), via inhibitory G-protein coupled receptors (GPCRs) (Trojanowski et al. 2015; Iannacone et al. 2017). Despite the identification of neuropeptides, only a small subset of GPCRs has been implicated in sleep regulation. The GPCR *dmsr-1* functions as a *flp-11* receptor on the RIS, which provides an autocrine feedback mechanism to regulate sleep duration, as well as a *flp-11* receptor on excitatory cholinergic neurons (Rossi et al. 2025), and a *flp-13* receptor on wake interneurons (Iannacone et al. 2017). The orphaned GPCR *npr-38* is required for sleep in the ADL sensory neurons and may play a broader timing role for SIS. Overexpression of *npr-38* by multicopy arrays causes a profound shift in the timing of sleep initiation, in that it occurs early when arousal and escape should dominate. Additionally, *npr-38* is required for heat and blue light avoidance behaviors, as well as general arousal (Le et al. 2023). Thus, *npr-38* may regulate the timing of ALA and RIS activation, together with other unknown pathways, to coordinate the broader stress response.

Only a small number of other cells have been implicated in SIS, which include ADL, AIY, RMG, DVA, and RIF, as well as cholinergic neurons. The AIY is one site of action for *dmsr-1* (Iannacone et al. 2017), and *dmsr-1* also functions in RIS, and cholinergic neurons (Rossi et al. 2025). RMG promotes arousal from SIS, possibly via the secretion of wake-promoting *pdf-1* neuropeptides (Choi et al. 2013; Soto et al. 2019). The neuropeptide receptor *npr-1* is required for SIS under certain conditions and functions in the RMG, thus RMG activation may override the ALA and RIS to promote arousal (Choi et al. 2013; Soto et al. 2019). The DVA and RIF also stimulate arousal; optogenetic activation of the cAMP pathway in either of these cells induces waking and impairs sleep. Also, cAMP levels are reduced in DVA and RIF during sleep (Cianciulli et al. 2019). The ADL, AIY, RMG, DVA, and RIF neurons modulate sleep in different ways, yet how they communicate with ALA and RIS mechanistically is still unclear.

Many questions surrounding SIS still remain not fully answered. First, how do animals coordinate the temporal aspects of sensory arousal with the opposing events of sleep neuron activation? This is an essential balance, considering animals must remove themselves from dangerous environments before entering a recovery sleep state, which will render them vulnerable to the initial threats. Next, how do sleep neurons mechanistically induce the behavioral aspects of SIS? Data suggest that during sleep, the activation of GPCRs leads to the mobilization of inhibitory G-proteins and thus the modulation of second messenger system; however, little mechanistic data have been shown to support such a model. The identification of new receptors and signaling molecules will be an important first step in expanding our mechanistic insight into these events. Last, what signals promote arousal from SIS, and how do they override the effects of sleep neuron activation?

**Fig. 1.**
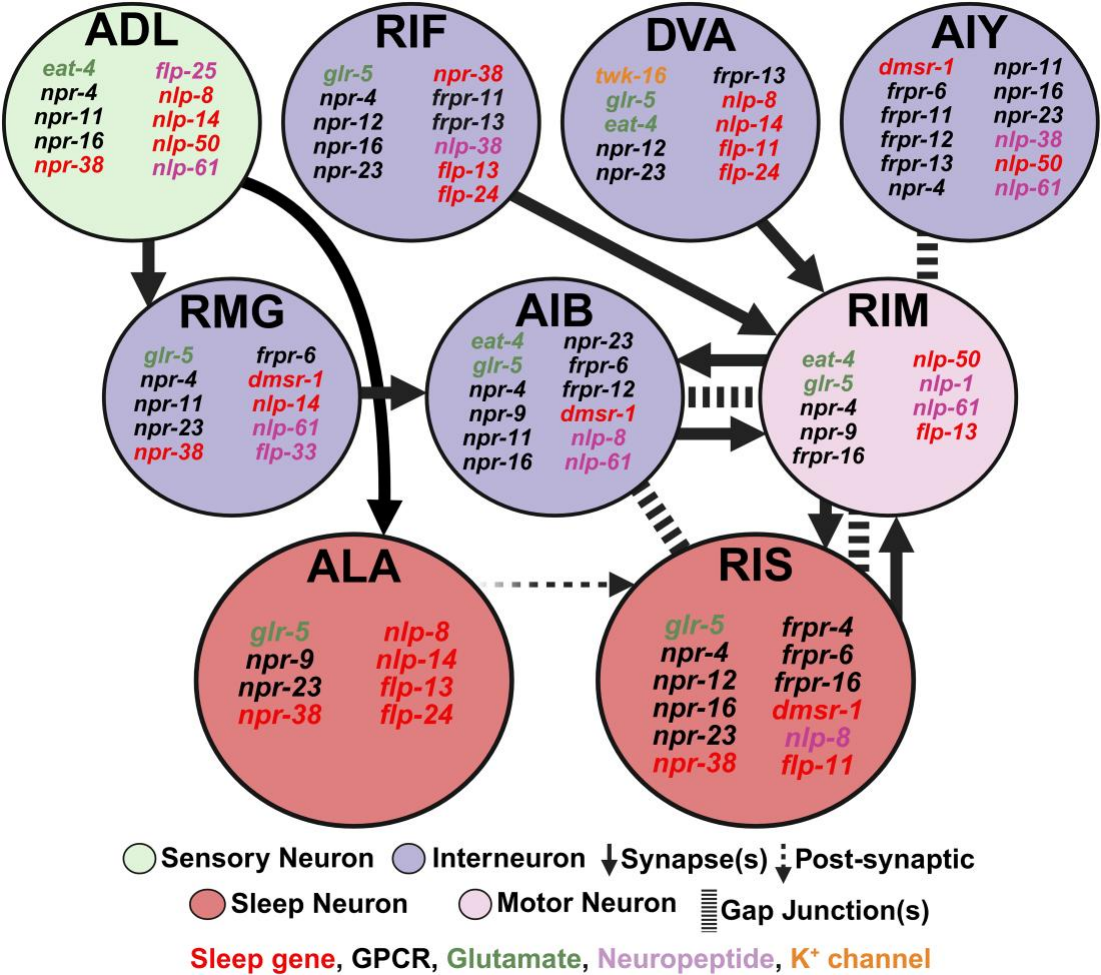
Expression of signaling molecule genes in a sleep-regulating circuit. Expression is based on published RNA-seq data (Taylor et al. 2021); chemical synapses are indicated by solid arrows, gap junctions by dashed lines (White et al. 1986), and postsynaptic communication by a dashed arrow (Konietzka et al. 2020). Created with BioRender.com.

As a first step to better understanding these mechanisms, we sought to identify new sleep-regulating molecules by measuring sleep in mutants of genes expressed in the abovementioned cells. Specifically, we focused on genes expressed in the ALA and RIS, considering their role as central sleep neurons, and their synaptic partners (Fig. 1) (White et al. 1986). We examined neuropeptides, GPCRs, a potassium channel expressed in a wake-promoting neuron, and the glutamate system (Fig. 1; Supplementary Table 1). Loss of function of some of the genes reduced sleep, while others enhanced it, and in some cases, we observed an alteration in the timing of sleep; a summary of the phenotypes can be found in Supplementary Table 2. We identified a particularly important role for the glutamate receptor *glr-5*, in which mutants displayed significantly less sleep and initiated sleep later than controls. Using cell-specific rescue, we propose a model in which *glr-5* functions in the RIS neuron to modulate sleep timing and in the RIS, AIB, and RIM neurons to regulate sleep amounts (i.e. sleep maintenance).

## Methods

### Worm maintenance and strains

Animals were maintained at 20 °C on agar plates containing nematode growth medium and fed the OP50 derivative bacterial strain DA837 (Davis et al. 1995). The strains that were used in this study were generated in the Nelson lab or obtained from the National BioResource Project, the *Caenorhabditis* Genetics Center, or the Raizen Lab (University of Pennsylvania) (Supplementary Table 1).

### Molecular biology and transgenesis

To generate DNA for transgenesis, polymerase chain reaction (PCR) or overlap extension-PCR (OE-PCR) was used (Nelson and Fitch 2011). Transgenic animals were made by microinjection (Stinchcomb et al. 1985). To make genomic rescue lines for *twk-16* and *glr-5*, and multicopy overexpression lines for *nlp-1* and *nlp-61*, the 5′UTR, coding sequence, and 3′UTR were amplified from wild-type genomic DNA for each corresponding gene. The *twk-16* wild-type amplicons were injected into *twk-16*(*stj13*) animals, and the *glr-5* wild-type PCR products were injected into *glr-5*(*stj554*) animals. PCR products of either *nlp-1* or *nlp-61* were injected into wild-type animals (N2). To generate cell-specific rescue constructs for *glr-5* and *npr-38*, and inducible overexpression constructs for *flp-3* and *flp-33*, OE-PCR was used. Constructs for cell-specific rescue were made by first amplifying the genomic promoter sequence of the genes *ver-3* (ALA) (Popovici et al. 2002; Van Buskirk and Sternberg 2010), *flp-11* (RIS) (Turek et al. 2013), *npr-9* (AIB) (Campbell et al. 2016), *aptf-1* (AIB + RIS) (Turek et al. 2013; Taylor et al. 2021), *flp-21* (RMG) (Kim and Li 2004), *nlp-6* (RIF), and *cex-1* (RIM) (Taylor et al. 2021). The corresponding promoter was fused by OE-PCR to the coding sequence and 3′UTR of *glr-5* or *npr-38*. To generate *flp-3* and *flp-33* overexpression constructs, the promoter from the gene *hsp-16.2* was fused to the coding sequence and 3′UTR of the corresponding gene by OE-PCR. Oligonucleotides are listed in Supplementary Table 3.

### Construction of mutants

The *nlp-61*, *frpr-4*, *frpr-11*, *frpr-12*, *frpr-13*, *twk-16*, and *glr-5* loss-of-function alleles were generated using a defined CRISPR approach (Paix et al. 2017). Single-stranded oligonucleotides composed of an insertion sequence containing stop codons and a NheI (New England Biolabs) restriction site, flanked by 35 bp homology arms, were constructed (Integrated DNA Technologies [IDT]) (Supplementary Table 3). For each CRISPR mutant, we simultaneously edited the *dpy-10* gene to allow for screening using the roller or dumpy physical phenotypes (Paix et al. 2017). A mixture of guide RNA duplexed with Alt-R CRISPR-Cas9 tracrRNA (IDT), Alt-R S.p. Cas9 Nuclease V3 (IDT), and oligonucleotide repair templates was injected into day-1 adult wild-type (N2) animals. For the *frpr-11*, *frpr-12*, and *frpr-13* mutants, we first made the *frpr-11*; *frpr-12* double mutant by injecting the CRISPR reagents for both genes into wild-type animals. Once these mutants were isolated, we then injected the *frpr-13* CRISPR mix into the double mutant. To identify all mutants, F1 animals displaying physical phenotypes (dumpy or roller) were singled, allowed to self, and screened by PCR followed by restriction digest. If the animals were homozygous for the *dpy-10* mutation, they were crossed with N2. For the AID-tagged *eat-4* mutant, we first PCR amplified the AID sequence from the plasmid pLZ29 (Addgene) (Zhang et al. 2015), using 2 different primer pairs, one with 100 bp homology arms and the other with 35 bp arms, which are complementary to the same sequence at the 3′ end of *eat-4*. These 2 PCR products were melted together to increase efficiency of insertion, as previously described (Ghanta and Mello 2020). The melted PCR product was used as the repair template to generate the strain SJU606. Candidate mutants were screened by PCR across the insertion site and identified based on size. All alleles were confirmed by Sanger sequencing (GENEWIZ).

### WorMotel behavioral assays

Movement quiescence for both developmentally timed sleep (DTS), and SIS was measured using the WorMotel, as previously described (Churgin et al. 2017). For SIS, L4 animals were picked the day prior to the experiment and allowed to grow to adulthood overnight. Day-1 adults were transferred to the agar surfaces of 24-well polydimethylsiloxane (PDMS) microchips and exposed to either UV irradiation, or noxious heat, and then imaged for 8 h. UV stress was applied by placing the entire microchip into a cross-linker (Ultraviolet, 254 UVP), which was set to 1500 J/m^2^ of UV light, as described (DeBardeleben et al. 2017). For heat stress, the microchip was placed in a petri dish, which was sealed with parafilm, and submerged in a 37 °C water bath for 30 min, and then imaged for 12 h. For each experiment, wild-type, and the relevant mutant, and/or transgenic animals were run on the same PDMS chip over multiple trials. For DTS, wild-type and mutant animals were placed on microchips during the L4 stage prior to entering lethargus and allowed to develop to young adulthood while being imaged over a 16-h time period. For both DTS and SIS, quiescence was quantified using pixel subtraction, as previously described (Yu et al. 2014; Churgin et al. 2017), and total movement quiescence over the duration of the sleep cycles was compared between genotypes using Student's *t*-test (wild-type and a single mutant) or 1-way ANOVA followed by Tukey's multiple comparisons test (wild type and 2 other genotypes). Temporal patterns were compared by 2-way ANOVA followed by Sidak's multiple comparisons test. Movement quiescence is one of a number of behavioral aspects of sleep; in each figure, sleep refers to movement quiescence only. Experiments were run on the benchtop within the Nelson lab, where ambient temperature can range from 20 to 25 °C based on the time of year, thus leading to variability in sleep. Statistical analyses were only made between genotypes that were run simultaneously on the same PDMS chip, but averaged over multiple trials that were run in a 1- to 2-wk span.

### Feeding rate of *eat-4* mutants

To measure pumping rate, day-1 adults were manually observed for 20-s intervals at 80× magnification using a stereomicroscope. A single pump was defined as one full movement of the pharyngeal grinder during both the contraction and relaxation phase (Raizen et al. 2012). For experiments involving auxin, the derivative indole-3-acetic acid (Thermo Fisher) was supplemented in the bacterial food, as described (Zhang et al. 2015), and pumping was measured after 5 or 24 h. Averages were calculated and compared by 1-way ANOVA followed by Tukey's multiple comparisons test.

### Acute quiescence upon heat exposure

We used manual observations to quantify the acute quiescence response to heat. For each experiment, 10 day-1 adults were transferred to standard growth plates, which were placed on a slide warmer set at 37 °C for 30 min. The number of animals that were quiescent for both moving and feeding was counted every 1, 5, or 10 min, depending on the experiment. This was repeated over multiple trials. The fraction of quiescence was calculated by dividing the number of quiescent worms by the total number of worms and then averaged across trials. Comparisons were made using 2-way ANOVA followed by Sidak's multiple comparisons.

### Avoidance response to blue light

To quantify blue light avoidance, body bends were counted manually over a 1-min span in day-1 adult animals, in the presence of directed blue light using a fluorescent stereomicroscope (Leica). A body bend was defined as one movement in either direction, in which the head changed direction. The average number of bends were calculated and compared across genotypes using 1-way ANOVA followed by Tukey's multiple comparisons test.

## Results

Numerous neuropeptide genes are expressed in this simple circuit (Fig. 1), and we focused on exploring sleep roles for a subset of them. First, *flp-3*, *flp-25*, and *flp-33* code RFamide peptides like *flp-11* and *flp-13* (Nathoo et al. 2001); RFamides play conserved sleep roles across phylogeny (Lenz et al. 2015; Nath et al. 2016; Turek et al. 2016; Lee et al. 2017). We focused on the genes *nlp-1*, *mihp-1* (also named *nlp-38*), and *nlp-61* due to their high levels of expression in some of these cells and because they were considered candidates as ligands for *npr-38* (Isabel Beets, personal communication). Loss-of-function alleles were available for each gene except *nlp-61*, which we constructed using CRISPR. We focused on UV-sleep because it is highly temporally reproducible, thus allows for the detection of timing defects.

### RFamide neuropeptides *flp-3*, *flp-13*, and *flp-33* promote sleep maintenance

**Fig. 2.**
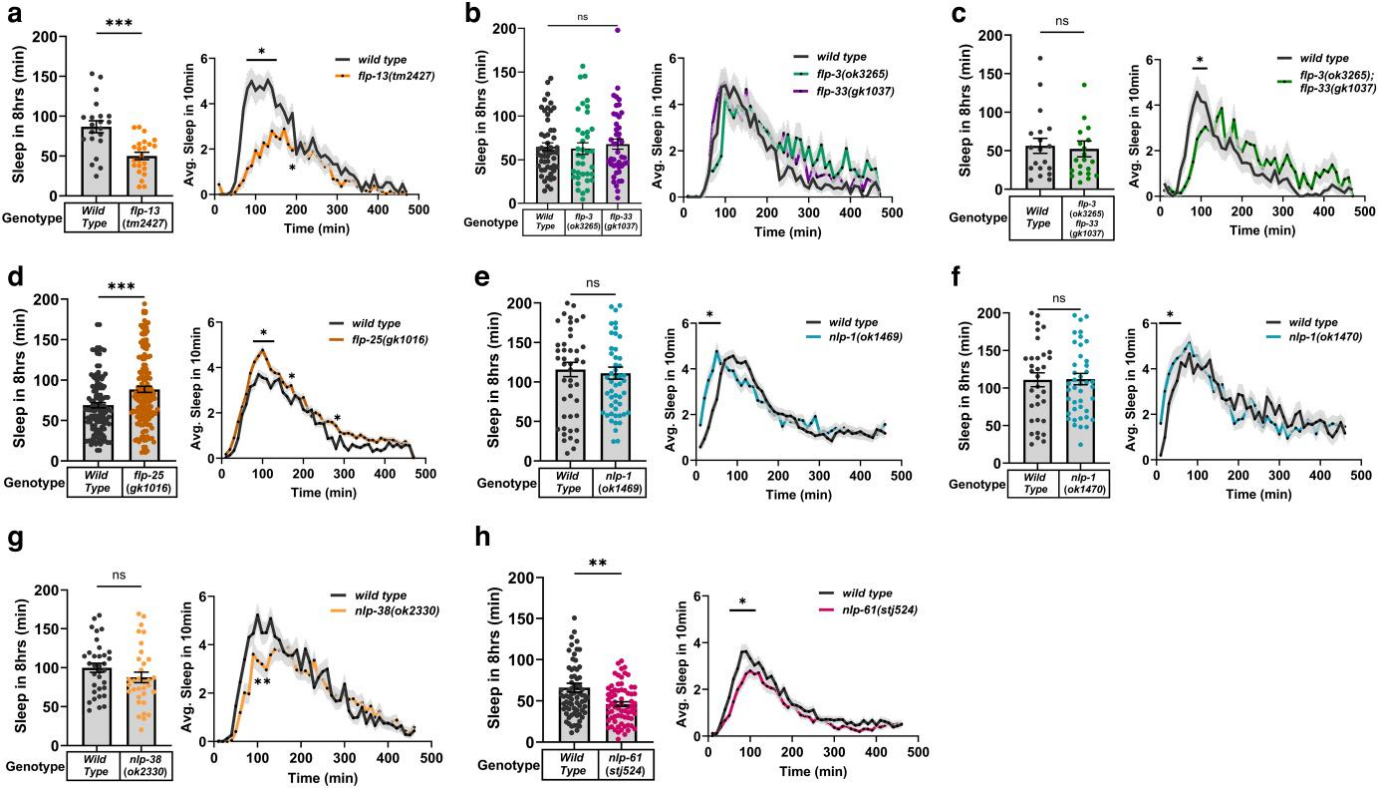
Multiple neuropeptide genes regulate SIS. For each panel, the average total minutes of movement quiescence during UV-induced sleep is displayed on the left, and the average minutes of movement quiescence in 10-min windows on the right. Sleep is defined as quiescence of movement, one prominent behavior of SIS. a) Quiescence in wild-type (*N* = 21) and *flp-13*(*tm2427*) (*N* = 23) animals. Right panel: *P < 0.05 at 70 to 140 and 190 min. b) Quiescence in wild-type (*N* = 53), *flp-3*(*ok3265*) (*N* = 37), and *flp-33*(*gk1037*) (*N* = 44) animals. c) Quiescence in wild-type (*N* = 19) and *flp-3*(*ok3265*); *flp-33*(*gk1037*) (*N* = 21) animals. Right panel: *P < 0.05 at 70 to 80 min. d) Quiescence in wild-type (*N* = 124) and *flp-25*(*gk1016*) (*N* = 171) animals. Right panel: *P < 0.05 at 80 to 120, 170, and 280 min. e) Quiescence in wild-type (*N* = 47) and *nlp-1*(*ok1469*) (*N* = 53) animals. Right panel: *P < 0.05 at 20 to 50 min. f) Quiescence in wild-type (*N* = 34) and *nlp-1*(*ok1470*) (*N* = 44) animals. Right panel: *P < 0.05 at 20 min only. g) Quiescence in wild-type (*N* = 34) and *nlp-38*(*ok2330*) (*N* = 33) animals. Right panel: *P < 0.05 at 80 and 100 min. h) Quiescence in wild-type (*N* = 74) and *nlp-61*(*stj524*) (*N* = 69) animals. Right panel: *P < 0.05 at 60, 80, and 90 min. Student's *t*-test was used for comparison between genotypes and 1-way ANOVA and Tukey's test for 3 genotypes. Temporal comparisons were made using 2-way ANOVA and Sidak's test (*P < 0.05, **P < 0.01, ***P < 0.001). Statistical analyses were performed using GraphPad Prism, and the figure was created with BioRender.com.

To validate the WorMotel system and establish a baseline for timing and sleep maintenance, we measured sleep in a known sleep-defective mutant, *flp-13*(*tm2427*). As expected, total sleep levels were significantly lower in *flp-13* mutants compared with wild type and reduced at multiple time points between 60 and 200 min post-UV exposure. Additionally, the peak of sleep induction was shifted to a later point than controls (Fig. 2a). Next, we measured UV-sleep in *flp-3* (expressed in PQR) and *flp-33* (Expressed in RMG) mutants, but did not observe any differences from wild type (Fig. 2b). However, *flp-3*; *flp-33* double mutants showed subtle alterations in sleep timing; the sleep program was delayed, with a significant difference in sleep at 70 and 80 min post-UV stress. The total amount of sleep was unchanged in these double mutants (Fig. 2c). These data suggest that *flp-3* and *flp-33* may function with *flp-13* to promote the onset of sleep.

### *flp-25* RFamide neuropeptides promote arousal

Next, we tested sleep in *flp-25* (expressed in ADL and PQG) mutants. Surprisingly, *flp-25* mutants displayed a significant increase in sleep (Fig. 2d). These data indicate that while a number of RFamide peptides positively regulate sleep, *flp-25* peptides promote arousal.

### *nlp-1* regulates sleep timing, and *nlp-38* and *nlp-61* promote sleep maintenance

Two different *nlp-1* (expressed in RIM) mutant strains were examined, and neither showed changes in total sleep; however, each displayed a shift in the timing of sleep (Fig. 2e and f). This early sleep phenotype is similar to that of *nlp-14* mutants and animals overexpressing *npr-38*. Thus, *nlp-1* may modulate sleep timing. Both *nlp-38* (expressed in AIY and RIF) and *nlp-61* (expressed in ADL, AIB, AIY, RIM, and RMG) mutants displayed reductions in sleep, but in different ways. Loss of *nlp-38* caused a small reduction in sleep at 80 and 100 min poststress but not in total levels (Fig. 2g). *nlp-61* mutants displayed a significant reduction in total sleep, with levels lower at 60, 80, and 90 min poststress (Fig. 2h). Thus, *nlp-38* and *nlp-61* may play small sleep-promoting roles.

### Overexpression of *flp-33* and *nlp-61* can induce movement quiescence

Ectopic overexpression of neuropeptide genes like *flp-11* and *flp-13* can induce sleeplike movement quiescence in active, awake adults. To determine if *flp-3* and *flp-33* can also induce quiescence of locomotion, we used a heat shock promoter to drive their expression in most cells in wild-type animals (Stringham et al. 1992). Two hours following a mild heat stress (33 °C), we manually measured body bends and found that while *flp-13* strongly reduced locomotion, *flp-33* only mildly reduced it, and *flp-3* did not reduce it at all (Supplementary Fig. 1a). Thus, *flp-33* is capable of reducing activity, but neither gene can induce a sleeplike state. Overexpression by transgenic multicopy arrays can also affect sleep, like we have shown previously with *nlp-50*; transgenic lines carrying additional copies of the *nlp-50* gene displayed a strong reduction in SIS (Le et al. 2023). We made overexpression lines for *nlp-1* and *nlp-61* and then measured UV-induced sleep and found that *nlp-1* overexpression did not alter SIS (Supplementary Fig. 1b), while *nlp-61* overexpression enhanced it (Supplementary Fig. 1c). Thus, *nlp-61* is required for sleep, and overexpression by multicopy array enhances it.

### *nlp-61* is required for DTS

To determine if either *nlp-1* or *nlp-61* is required for DTS, we measured L4 DTS in *nlp-1* mutants, but detected no differences from wild-type animals (Supplementary Fig. 2a); however, *nlp-61* mutants showed a significant reduction (Supplementary Fig. 2b). Thus, *nlp-61* is also required for DTS and thus may play a broader role in sleep regulation.

### *nlp-1* and *nlp-61* are not endogenous ligands for the GPCR *npr-38*

The orphaned GPCR *npr-38* is required for SIS; loss of function of *npr-38* reduces sleep and disrupts avoidance behavior, while *npr-38* overexpression profoundly shifts the timing of sleep to an earlier period. Where in the nervous system *npr-38* is functioning to coordinate the timing of sleep is unknown. *npr-38* is expressed in all of the cells of this circuit (Fig. 1), except AIY. Cell-specific rescue of *npr-38* in some of the cells has been examined (RIS, RMG, ALA, AIB, ADL, and DVA); however, only expression in the ADL restored sleep, but did not alter the timing of sleep (Le et al. 2023). To test the other cells in this circuit, we restored *npr-38* in the *npr-38*(*stj367*) background in the RIF (promoter: *nlp-6*) and RIM (promoter: *cex-1*) and measured UV-induced sleep. RIF expression did not affect sleep in any noticeable manner (Supplementary Fig. 3a), while expression in RIM restored sleep amounts, but did not alter the timing of sleep (Supplementary Fig. 3b). These data indicate that *npr-38* can function in ADL and RIM to regulate sleep amounts, but how it coordinates the temporal aspects of sleep is still unknown.

Overexpression phenotypes of neuropeptides require their downstream receptors, like what was observed with *flp-13* and *dmsr-1* (Iannacone et al. 2017), and phenotypes caused by multicopy overexpression of GPCRs require the presence of endogenous ligands. This was demonstrated with the GPCR *egl-6* and ligands *flp-10*/*flp-17* in the egg-laying circuit (Ringstad and Horvitz 2008) and the GPCR *frpr-4* and *flp-13* during body bend control (Nelson et al. 2015). *nlp-1* and/or *nlp-61* were potential candidate ligands for *npr-38* (Isabel Beets, personal communication). If *nlp-61* peptides are ligands for *npr-38 in vivo*, then *npr-38* should be required for the enhanced sleep of *nlp-61* overexpression (Supplementary Fig. 1c), and *nlp-61* should be required for *npr-38* overexpression phenotypes. We found that *nlp-61* overexpression animals did indeed sleep significantly less in the absence of *npr-38*, compared with controls (Supplementary Fig. 3c). However, *npr-38* overexpression in the *nlp-61*(*stj524*) background did not affect the temporal pattern of sleep (Supplementary Fig. 3d). These data do not support *npr-38* functioning as a receptor for *nlp-61*, but instead suggest that *npr-38* functions downstream of *nlp-61*. Next, we measured sleep in animals that overexpressed *npr-38* in the *nlp-1*(*ok1469*) background and found a modulation of the sleep phenotype but not a requirement for the *nlp-1* peptides. Specifically, animals slept early, but the amount of sleep was higher in *nlp-1* mutants (Supplementary Fig. 3e). What these genetic interactions indicate at the mechanistic level is not clear; however, these data do not support *nlp-1* function as ligands for *npr-38*.

### *nlp-1* mutants display timing defects during acute heat exposure

*npr-38* overexpression animals display behavioral quiescence rapidly when exposed to acute heat stress, significantly earlier than wild-type controls (Supplementary Fig. 3f). We found that *nlp-1* mutants displayed early UV-induced sleep (Fig. 2e), and in addition displayed behavioral quiescence faster on heat (Supplementary Fig. 3g). Taken together, we propose that *npr-38* and *nlp-1* coordinate the timing of sleep, and our data indicate that they may function in parallel.

### The GPCR *frpr-4* suppresses sleep maintenance

**Fig. 3.**
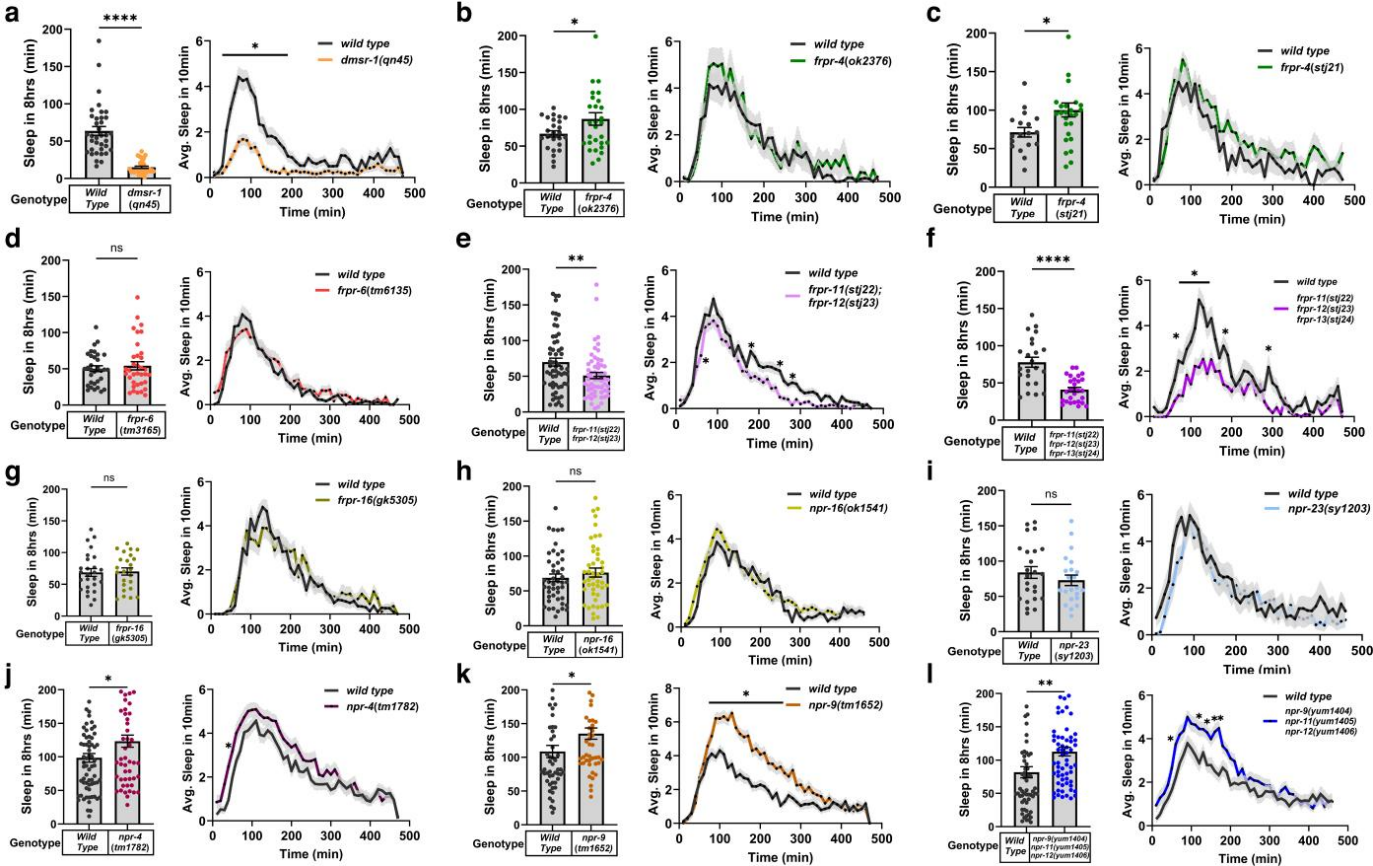
Multiple GPCR genes regulate SIS. For each panel, the total minutes of movement quiescence (left) and average minutes of movement quiescence during UV-induced sleep in 10-min windows (right) are displayed. Sleep is defined as quiescence of movement, one prominent behavior of SIS. a) Quiescence in wild-type (*N* = 36) and *dmsr-1*(*qn45*) (*N* = 36) animals. Right panel: *P < 0.05 at 40 to 170 min. b) Quiescence in wild-type (*N* = 26) and *frpr-4*(*ok2376*) (*N* = 29) animals, and c) wild-type (*N* = 18) and *frpr-4*(*stj21*) (*N* = 27) animals. d) Quiescence in wild-type (*N* = 32) and *frpr-6*(*tm6135*) (*N* = 35) animals. e) Quiescence in wild-type (*N* = 59) and *frpr-11*(*stj22*); *frpr-12*(*stj23*) (*N* = 57) animals. Right panel: *P < 0.05 at 60, 180, 250, and 280 min. f) Quiescence in wild-type (*N* = 25) and *frpr-11*(*sjt22*); *frpr-12*(*stj23*); *frpr-13*(*stj24*) (*N* = 28) animals. Right panel: *P < 0.05 at 70, 100 to 150, 180, and 290 min. g) Quiescence in wild-type (*N* = 26) and *frpr-16*(*gk5305*) (*N* = 24) animals. h) Quiescence in wild-type (*N* = 46) and *npr-16*(*ok1541*) (*N* = 46) animals. i) Quiescence in wild-type (*N* = 24) and *npr-23*(*sy1203*) (*N* = 22) animals. j) Quiescence in wild-type (*N* = 69) and *npr-4*(*tm1782*) (*N* = 53) animals. Right panel: *P < 0.05 at 40 min. k) Quiescence in wild-type (*N* = 52) and *npr-9*(*tm1652*) (*N* = 48) animals. Right panel: *P < 0.05 at 90 to 260 min. l) Quiescence in wild-type (*N* = 62) and *npr-9(yum1404); npr-11(yum1405)*; *npr-12(yum1406)* (*N* = 65) animals. Right panel: *P < 0.05 at 60, 120, 140, 160 to 170 min. Student's *t*-test was used to statistically compare the total quiescence of 2 genotypes (*P < 0.05, **P < 0.01, ****P < 0.0001), and temporal comparisons between genotypes were made using 2-way ANOVA followed by Sidak's test. Statistical analyses were performed using GraphPad Prism, and the figure was created with BioRender.com.

Numerous neuropeptide GPCR genes are expressed in this circuit, which include FMRFamide peptide receptor (*frpr*) and neuropeptide receptor (*npr*) genes (Fig. 1), of which we focused on highly expressed ones. We first measured UV-sleep in *dmsr-1* (expressed in AIY and RIS) mutants, well-established sleep-defective animals. *dmsr-1*(*qn45*) mutants displayed severely reduced SIS compared with wild-type controls, yet the timing of sleep appeared to be mostly unaltered (Fig. 3a). Next, we measured sleep in *frpr-4* (expressed in RIS); our previous work demonstrated that *frpr-4* is a *flp-13* receptor and that *frpr-4* overexpression induces behavioral quiescence (Nelson et al. 2015). However, *frpr-4* deletion mutants (allele *ok2376*) did not display significant defects in heat-induced sleep (Nelson et al. 2015). Here, we measured UV-induced sleep, and surprisingly, *frpr-4*(*ok2376*) animals displayed a significant increase in sleep, with no changes in timing (Fig. 3b). Considering our prior focus on *frpr-4*, we constructed a second loss-of-function mutant using CRISPR, *frpr-4*(*stj21*), and again found that sleep was significantly enhanced (Fig. 3c). Thus, *frpr-4* signaling may function to negatively regulate sleep maintenance, but not timing.

### The paralogs *frpr-11*, *frpr-12*, and *frpr-13* promote sleep maintenance

*frpr-6* (expressed in AIY, RIS, and RMG), *frpr-11* (expressed in AIY and RIF), *frpr-12* (expressed in AIB and AIY), *frpr-13* (expressed in AIY, DVA, and RIF), and *frpr-16* (expressed in RIM and RIS) are potential paralogs with *frpr-4* and expressed in this circuit. *frpr-6* is the most similar to *frpr-4*; however, deletion mutants did not display sleep defects (Fig. 3d). *frpr-11*, *frpr-12*, and *frpr-13* are highly similar to one another and are located adjacently on chromosome V, with *frpr-11* and *frpr-12* being almost identical, indicating they likely arose by gene duplication (McCoy et al. 2014). We constructed an *frpr-11*; *frpr-12* double mutant and measured a modest but significant reduction in total levels of SIS, as well as at various time points throughout sleep (Fig. 3e). Next, we generated an *frpr-11*; *frpr-12*; *frpr-13* triple mutant, and found that sleep was even more reduced (Fig. 3f), with the timing mostly unaltered. These data indicate that *frpr-11*, *frpr-12*, and *frpr-13* function redundantly to positively regulate sleep maintenance.

### The GPCRs *npr-4* and *npr-9* suppress sleep maintenance

Next, we measured sleep in *frpr-16*, *npr-16* (expressed in ADL, AIB, AIY, RIF, and RIS), and *npr-23* (expressed in AIB, AIY, ALA, DVA, RIF, RIS, and RMG) mutants; however, we did not detect any changes in sleep (Fig. 3g to i). However, when we measured sleep in *npr-4* (expressed in ADL, AIB, RIF, RIM, RIS, and RMG) and *npr-9* (expressed in AIB, ALA, and RIM) mutants, sleep was significantly enhanced. Total sleep was increased in both mutants; sleep occurred slightly earlier in *npr-4* animals, while sleep was enhanced at numerous time points during the normal sleep cycle in *npr-9* mutants (Fig. 3j and k). We also measured sleep in *npr-9*;*npr-11*;*npr-12* triple mutants (*npr-11* is expressed in ADL, AIB, AIY, and RMG; *npr-12* is expressed in DVA, RIF, and RIS) (Pu et al. 2023), and found that sleep was enhanced, but occurred slightly earlier than controls (Fig. 3l). Total levels did not appear to be higher than *npr-9* single mutants. Overall, these data suggest that *npr-4* and *npr-9* negatively regulate sleep maintenance and potentially sleep timing. More broadly, numerous GPCRs may modulate sleep in unique and opposing ways, indicating complex antagonistic signaling mechanisms modulate sleep maintenance and timing.

### The 2-pored potassium channel *twk-16* negatively regulates sleep

**Fig. 4.**
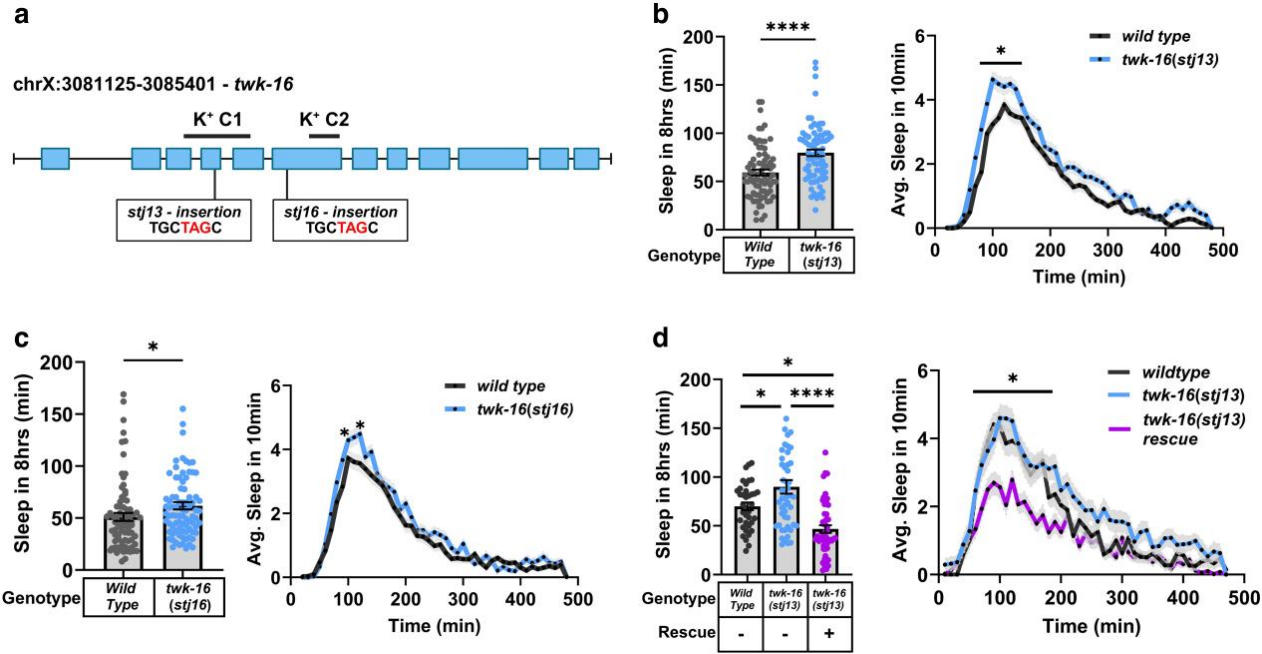
*twk-16* loss-of-function enhances sleep. a) Schematic of the gene and allele structure for the 2-pored potassium channel gene *twk-16*. K^+^C1, potassium channel 1; K^+^C2, potassium channel 2. For b) to d), sleep is defined as movement quiescence during UV-induced sleep. b) Quiescence in wild-type (*N* = 77) and *twk-16*(*stj13*) (*N* = 76) animals. Right panel: *P < 0.05 at 60 to 100 and 120 min. c) Quiescence in wild-type (*N* = 79) and *twk-16*(*stj16*) (*N* = 84) animals. Right panel: *P < 0.05 at 80 and 120 min. d) Quiescence in wild-type (*N* = 37), *twk-16*(*stj13*) (*N* = 51), and *twk-16*(*stj13*) transgenic animals expressing wild-type *twk-16* copies (*N* = 51). Right panel: Comparing *twk-16*(*stj13*) with rescued strain, *P < 0.05 at 60 to 230 min. Student's *t*-test was used to statistically compare 2 genotypes, 1-way ANOVA followed by Tukey's test to compare 3 genotypes, and temporal comparisons were made using 2-way ANOVA followed by Sidak's test (*P < 0.05, ****P < 0.0001). Statistical analyses were performed using GraphPad Prism, and the figure was created with BioRender.com.

The gene *twk-16*, which codes a mechanically gated, 2-pored weakly inward rectifying potassium channel (Salkoff et al. 2001; Das et al. 2021), is selectively expressed in the DVA (Puckett Robinson et al. 2013). *twk-16* may regulate DVA membrane potential, which regulates body wall muscles and bending angle. The role of DVA during sleep regulation is unclear; however, activation of a red-light-induced adenylyl cyclase called IlaC22 (Ryu et al. 2014) in the DVA impairs SIS (Cianciulli et al. 2019), suggesting it is wake promoting. To further explore this, we generated 2 loss-of-function alleles, *stj13* and *stj16* (Fig. 4a). Both *twk-16*(*stj13*) and *twk-16*(*stj16*) animals displayed modest but significant enhancement of sleep, with the peak of sleep between 90 and 120 min poststress being consistently higher than controls (Fig. 4b and c); however, the timing of sleep was unaffected. Next, we made a transgenic multicopy rescue strain, in which wild-type *twk-16* genes driven from their endogenous promoter were expressed in the *twk-16*(*stj13*) background. This rescue of *twk-16* caused a significant impairment of total sleep (Fig. 4d). These data suggest that *twk-16* negatively regulates sleep maintenance, possibly by maintaining resting potential of the wake-promoting DVA neuron. *twk-16* is also expressed in the AS, AVK, AVH, DVC, AS11, AUA, AVB, and RIC neurons; thus, we cannot rule out a role for these cells during sleep regulation as well.

### *eat-4* and *glr-5* are required for SIS maintenance and timing

**Fig. 5.**
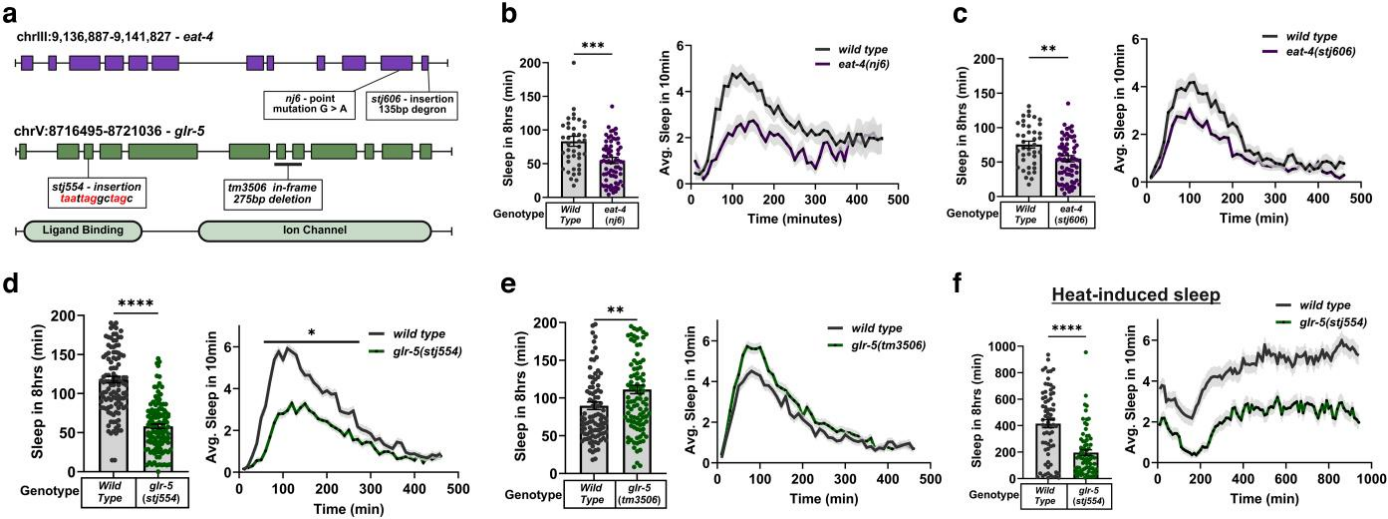
Glutamate signaling is required for SIS. a) Schematic of the gene and allele structure for *eat-4* and *glr-5*. For b) to f), the total minutes of movement quiescence (left) and average minutes of movement quiescence in 10-min windows (right) are displayed. b) Quiescence during UV-induced SIS in wild-type (*N* = 46) and *eat-4*(*nj6*) (*N* = 31) animals. c) Quiescence during UV-induced SIS in wild-type (*N* = 40) and *eat-4*(*stj606*) (*N* = 71) animals. d) Quiescence during UV-induced SIS in wild-type (*N* = 113) and *glr-5*(*stj554*) (*N* = 121) animals. Right panel: *P < 0.05 at 40 to 240 min, 2-way ANOVA followed by Sidak's test. e) Quiescence during UV-induced SIS in wild-type (*N* = 99) and *glr-5*(*tm3506*) (*N* = 105) animals. f) Quiescence during heat-induced SIS in wild-type (*N* = 113) and *glr-5*(*stj554*) (*N* = 121) animals. Student's *t*-test was used to statistically compare sleep totals (**P < 0.01, ***P < 0.001, ****P < 0.0001). For b) to f), sleep is defined as quiescence of movement. Statistical analyses were performed using GraphPad Prism, and the figure was created with BioRender.com.

The vesicular glutamate transporter gene *eat-4* (Lee et al. 1999) (expressed in ADL, AIB, DVA, and RIM) and the non-N-methyl-D-aspartate glutamate receptor *glr-5* (Brockie et al. 2001) (expressed in AIB, ALA, DVA, RIF, RIM, RIS, and RMG) are expressed in a number of cells in this circuit (Fig. 1). So, we measured sleep in *eat-4*(*nj6*) loss-of-function mutants (Ohnishi et al. 2011) and found that SIS was reduced (Fig. 5b), suggesting that glutamate positively regulates SIS. Next, we used CRISPR to tag the 3′ end of *eat-4* with a degron sequence (*stj606* allele), to allow for the degradation of EAT-4 protein using the auxin-inducible degradation (AID) system (Zhang et al. 2015). However, feeding rate (i.e. pharyngeal pumping), a phenotype known to be impaired in *eat-4* loss-of-function mutants (Lee et al. 1999; Dalliere et al. 2016), was reduced prior to conditional degradation (Supplementary Fig. 4a). Expression of the plant gene *tir1* (Zhang et al. 2015), in all cells using the *eft-3* promoter, followed by auxin treatment for 5 or 24 h did not further reduce pumping (Supplementary Fig. 4b). We conclude that the degron tag at the 3′ end of *eat-4* results in a hypomorphic allele and is not useful for the AID system. Despite this, we measured sleep in *eat-4*(*stj606*) animals in the absence of auxin and found sleep to be reduced but appeared less severe than *eat-4*(*ky5*) animals (Fig. 5c). Taken together, these data suggest that glutamate signaling positively regulates SIS.

Next, we measured sleep in 2 *glr-5* mutants, *glr-5*(*tm3506*), which contain a small in-frame deletion, and *glr-5*(*stj554*), who carry an insertion of 3 stop codons in each reading frame in the third exon of the gene, presumably a null (Fig. 5a). We found that *glr-5*(*stj554*) animals displayed impaired sleep; it was initiated later and lower overall (Fig. 5d**)**. These data suggest that *glr-5* promotes the initiation and maintenance of sleep. Considering that UV-sleep was so impaired in these animals, we wanted to test if *glr-5* was required for other forms of sleep. Heat-induced sleep and L4 DTS were also reduced in these mutants (Fig. 5f; Supplementary Fig. 2d). Additionally, we measured the acute response to noxious heat and found that animals displayed increased activity compared with controls (Supplementary Fig. 5a and b). We also assessed their response to directed blue light, a noxious stimulant known to initiate avoidance (Edwards et al. 2008), and found that *glr-5* mutants responded slower than controls (Supplementary Fig. 5c). Thus, *glr-5* is required for behaviors of the stress response, including sensory responsiveness to noxious stimuli, sleep timing, and sleep maintenance.

*glr-5* (*tm3506*) mutants have egg-laying defects (Wen et al. 2020) and an impaired response to chemical repellants (Zou et al. 2018). Surprisingly, UV-induced sleep was modestly but significantly enhanced in these animals (Fig. 5e). While the *stj554* allele likely results in a severely truncated protein that lacks a glutamate binding domain and ion channel, thus is loss of function, the *tm3506* allele is a small 275-bp deletion in the ion channel coding sequence. This would truncate the ion channel domain by 43 amino acids (Fig. 5a; Supplementary Fig. 6b); however, it is unclear if this would impair ion channel function or lead to a constitutively open channel. First, we sought to confirm the opposing sleep phenotypes of these mutants by analyzing *glr-5*(*stj554*) and *glr-5*(*tm3506*) animals side-by-side in the same experiments. As expected, *glr-5*(*stj554*) animals displayed reduced sleep, while *glr-5*(*tm3506*) animals slept significantly more (Supplementary Fig. 6a). Based on this, we conclude that *tm3506* disrupts *glr-5* function and may lead to a gain-of-function phenotype caused by constitutive activation of the ion channel domain.

### *glr-5* functions in the ALA, RIS, AIB, and RIM to control sleep timing and amounts

**Fig. 6.**
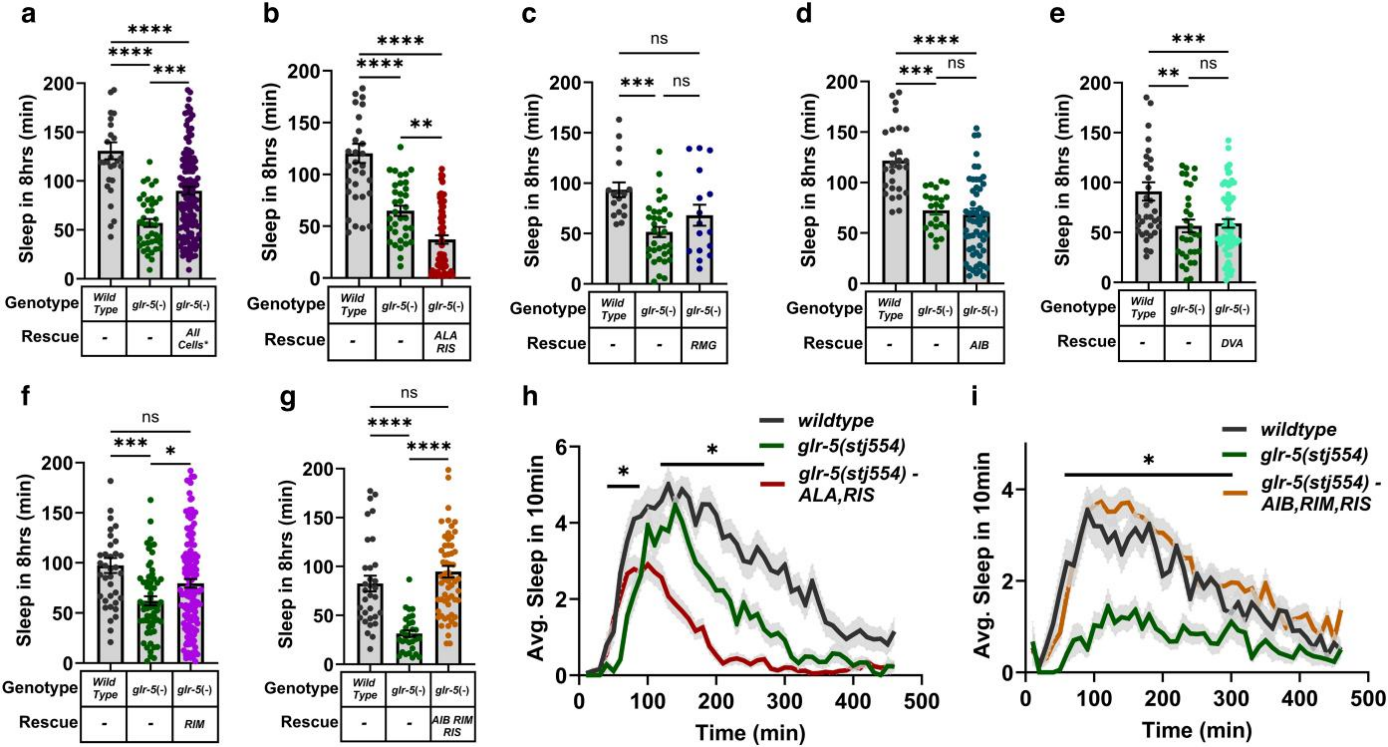
*glr-5* functions in the AIB, RIM, and RIS to regulate UV-induced sleep. a) Quiescence during UV-induced SIS in wild-type (*N* = 29), *glr-5*(*stj554*) (*N* = 41), and transgenic *glr-5*(*stj554*) (*N* = 68) (Rescue: *all cells in which *glr-5* is expressed; Promoter: *glr-5*) animals. b) Quiescence during UV-induced SIS in wild-type (*N* = 32), *glr-5*(*stj554*) (*N* = 34), and transgenic *glr-5*(*stj554*) (*N* = 65) (Rescue: ALA and RIS; Promoters: *ver-3* and *flp-11*) animals. c) Quiescence during UV-induced SIS in wild-type (*N* = 17), *glr-5*(*stj554*) (*N* = 34), and transgenic *glr-5*(*stj554*) (*N* = 16) (Rescue: RMG; Promoter: *flp-21*) animals. d) Quiescence during UV-induced SIS in wild-type (*N* = 27), *glr-5*(*stj554*) (*N* = 23), and transgenic *glr-5*(*stj554*) (*N* = 58) (Rescue: AIB; Promoter: *npr-9*) animals. e) Quiescence during UV-induced SIS in wild-type (*N* = 35), *glr-5*(*stj554*) (*N* = 32), and transgenic *glr-5*(*stj554*) (*N* = 63) (Rescue: DVA; Promoter: *twk-16*) animals. f) Quiescence during UV-induced SIS in wild-type (*N* = 36), *glr-5*(*stj554*) (*N* = 30), and transgenic *glr-5*(*stj554*) (*N* = 60) (Rescue: RIM; Promoter: *cex-1*) animals. g) Quiescence during UV-induced SIS in wild-type (*N* = 32), *glr-5*(*stj554*) (*N* = 30), and transgenic *glr-5*(*stj554*) (*N* = 61) (Rescue: AIB, RIM, RIS; Promoters: *aptf-1* and *cex-1*) animals. For a) to g), statistical significance was calculated by 1-way ANOVA followed by Tukey's test (**P < 0.01, ***P < 0.001, ****P < 0.0001). h) Average quiescence in 10-min windows in wild-type (*N* = 32), *glr-5*(*stj554*) (*N* = 34), and transgenic *glr-5*(*stj554*) (*N* = 65) (Rescue: ALA and RIS; Promoters: *ver-3* and *flp-11*) animals. i) Average quiescence in 10-min windows in wild-type (*N* = 32), *glr-5*(*stj554*) (*N* = 30), and transgenic *glr-5*(*stj554*) (*N* = 61) (Rescue: AIB, RIM, RIS; Promoters: *aptf-1* and *cex-1*) animals. Comparisons were made using 2-way ANOVA followed by Sidak's test (*P < 0.05). For each panel, sleep is defined as quiescence of movement. Statistical analyses were performed using GraphPad Prism, and the figure was created with BioRender.com.

We next sought to determine in which cells *glr-5* functions during sleep. We constructed transgenic *glr-5*(*stj554*) animals that expressed the wild-type *glr-5* from various promoters expressed in AIB (*npr-9*), ALA (*ver-3*), DVA (*twk-16*), RIM (*cex-1*), RIS (*flp-11* or *aptf-1*), and RMG (*flp-21*), as well as from the *glr-5* endogenous promoter (genomic rescue). Genomic rescue of *glr-5* increased sleep close to wild-type levels (Fig. 6a). We speculate that the lack of full rescue could be due to dosage effects of the multicopy array. Considering ALA and RIS are central sleep neurons, we rescued *glr-5* in the ALA and RIS together and then individually in RMG, AIB, or DVA; however, sleep was not restored under any of these conditions (Fig. 6b to e). In fact, expression in the ALA and RIS together further reduced sleep lower than *glr-5* mutants alone (Fig. 6b). However, there was a striking rescue of the temporal defects of *glr-5* mutants (Fig. 6h). These data suggest that sleep initiation (i.e. timing) is regulated by *glr-5* through the RIS and/or ALA. The observation that sleep is further reduced when *glr-5* is rescued in the ALA and RIS may indicate that *glr-5* can positively and negatively regulate sleep neurons. However, we cannot rule out technical explanations, such as high expression levels common with multicopy rescue experiments, or genetic interactions caused by the promoters that were employed in these experiments. The RIS is highly connected to the AIB and RIM interneurons by gap and chemical junctions (Fig. 1) (White et al. 1986). Rescue in the RIM modestly increased sleep levels but did not alter the timing of sleep (Fig. 6f); however, expression in the AIB, RIM, and RIS restored sleep to wild-type levels and rescued the timing defects (Fig. 6g and i). We propose a model in which *glr-5* functions to regulate the sleep timing through the RIS and maintenance through AIB, RIS, and RIM, which form a highly connected 3-celled circuit (Supplementary Fig. 7).

## Discussion

Transitions between sleep and arousal are coordinated by antagonistic action of sleep- and wake-active interneurons, which orchestrate global brain states (Nichols et al. 2017). In mammals, sleep is dominated by the action of GABAergic and neuropeptidergic sleep neurons (Saper et al. 2010; Bringmann 2018). Their activation during daily sleep of insects and mammals, and DTS of nematodes, is influenced by circadian input and homeostasis (Saper et al. 2005; Monsalve et al. 2011). As homeostatic sleep drive builds throughout the day, one function of the clock may be to ensure that arousal is maintained, and sleep initiation is repressed until an animal can safely rest (Shafer and Keene 2021). If sleep pressures are enhanced outside of normal daily conditions, like during times of injury and infection, this can override circadian control and induce sleep during the day or heighten it at night (Imeri and Opp 2009; Davis and Raizen 2017). SIS of invertebrates has become an important model for exploring the mechanisms of sleep outside of circadian regulation, but also for understanding how sleep and arousal are balanced by organisms (Hill et al. 2014; Lenz et al. 2015). Here, we used *C. elegans* SIS as a model to identify new signaling molecules that regulate sleep timing and maintenance. We have found genes that both positively and negatively regulate sleep timing and maintenance. In particular, we find that glutamate signaling, and a number of neuropeptides and GPCRs, including *nlp-38*, *nlp-61*, *flp-3*, *flp-33*, *frpr-11*, *frpr-12*, *and frpr-13* positively regulate sleep, while *twk-16*, *frpr-4*, *flp-25*, *npr-4*, and *npr-9* act as negative regulators. The identification of these opposing signals is an important first step in better understanding how sleep and arousal are balanced.

Our data support the hypothesis that mechanisms have evolved to negatively and positively regulate sleep initiation and sleep depth (i.e. maintenance). This is not overly surprising, considering selective evolutionary pressures exist for and against sleep. Sleep is essential, yet renders animals vulnerable to predation and limits foraging and reproduction (Keene and Duboue 2018; Anafi et al. 2019). The opposing mechanisms that underlie sleep and arousal, both at the genetic and cellular level, need further study, and the SIS model of *C. elegans* provides a unique avenue for mechanistic dissection.

Upon threat exposure, *C. elegans* will initiate stereotypical avoidance responses, consisting of accelerated movement and heightened arousal (Culotti and Russell 1978). Injury requires recovery sleep for survival; however, sleep must be precisely timed in order to prevent further damage. The ALA and RIS neurons are required for sleep, their depolarization induces sleep, and their activation correlates with sleep in real-time (Turek et al. 2013; Hill et al. 2014; Nelson et al. 2014; Konietzka et al. 2020). As with sleep in other animals, opposing mechanisms must be in place to coordinate sleep and arousal. Our prior work identified the molecules *nlp-14* (expressed in ALA) and *npr-38* as candidates for coordinating these behaviors. *nlp-14* mutants and *npr-38* overexpressing animals display early sleep, and *npr-38* mutants show moderate delays in SIS (Honer et al. 2020; Le et al. 2023), suggesting that *nlp-14* and *npr-38* negatively and positively regulate sleep initiation, respectively. The *nlp-14* receptor and *npr-38* ligands, as well as an understanding of the timing mechanisms, are unknown. This motivated our current study, where we sought to identify new sleep-regulatory genes expressed in ALA, RIS, and/or a subset of cells connected with them (Fig. 1). Using this approach, we identified signaling molecules that both positively and negatively regulate sleep timing and sleep maintenance, which will allow for future mechanistic studies using the SIS model of *C. elegans*.

### Sleep timing

The timing of sleep neuron activation is likely a crucial component of the temporal dynamics of these events. During times of sensory arousal, molecules that suppress sleep initiation will dominate; however, as cells incur damage, they will release *siss-1* epidermal growth factors, which signal to the ALA and RIS, through the *let-23* receptor and second messenger pathways controlled by phospholipase C (Van Buskirk and Sternberg 2007; Hill et al. 2024). Controlled doses of UV irradiation can induce sleep that is highly experimentally reproducible, such that animals remain active for ∼30 to 45 min prior to sleep induction (DeBardeleben et al. 2017). Genetic manipulations that cause sleep to occur earlier involve negative regulators of sleep induction, while those that shift sleep to later timepoints are positive regulators. In this current study, we have identified both. *nlp-1* mutants initiate sleep early, thus *nlp-1* peptides may act as negative regulators of sleep induction. They are expressed in the RIM, which is presynaptic to RIS, thus could function to inhibit RIS as sleep drive increases following injury. We propose a model in which *nlp-1* functions in parallel with *nlp-14* to prevent sleep initiation during arousal (Supplementary Fig. 7a). Loss of *flp-3* and *flp-33*, *flp-13*, *nlp-61*, or *glr-5* initiates sleep later, while overexpression of *npr-38* causes early sleep; thus, these genes may represent positive regulators of sleep. However, overexpression of *nlp-61* does not result in an early sleep phenotype. These data indicate that for sleep timing, requirement of a gene does not necessarily correlate with sufficiency. Even in the context of these mutations, sleep eventually initiated, presumably via *siss-1*-mediated activation of ALA and RIS. However, we again propose a model where multiple signals function at the level of sleep neurons to allow for robust activation at the correct time (Supplementary Fig. 7a). Our rescue data, in particular, indicate that *glr-5* functions directly on RIS to positively regulate sleep initiation (Fig. 6h). Future work will be focused on identifying the sites of action for the other molecules identified here.

### Sleep maintenance

Multiple mutations that enhance or impair sleep in *Drosophila* (Shafer and Keene 2021), mammals (Shen et al. 2022), and *C. elegans* (Honer and Nelson 2024) have been identified, indicating complex genetic redundancy regulates depth (i.e. maintenance) of sleep. During SIS of *C. elegans*, the RIS sleep neuron itself can in part regulate the duration and depth of sleep, by inhibitory autocrine signaling by *flp-11*/*dmsr-1* (Rossi et al. 2025). While other peptides released from ALA and RIS are believed to contribute downstream of the sleep neurons. Here, we measured sleep in signaling molecules expressed in cells that are upstream of ALA and RIS, thus may play more of a role in the maintenance of sleep neuron activation. The molecules identified here that may function as positive regulators of sleep maintenance include *nlp-38*, *nlp-61*, *flp-3*, *flp-33*, *frpr-11*, *frpr-12*, *frpr-13*, *eat-4*, and *glr-5*. Significant genetic redundancy may exist to promote sleep, a hypothesis supported by our data relating to *frpr-11*, *frpr-12*, and *frpr-13*. While the specific *in vivo* ligands of these receptors are still unknown, both *frpr-11* and *frpr-13* were activated in cell culture systems by the same ligands and have been classified as promiscuous GPCRs, which are activated by numerous *flp* encoding peptides (Beets et al. 2023). Interestingly, these include the sleep-promoting *flp-11* and *flp-13* peptides (Nelson et al. 2014; Turek et al. 2016). This may suggest that sleep-promoting peptides activate a collection of GPCRs, which allow them to silence wake neurons more robustly, or this may potentially provide a mechanism for eliciting their cellular effects in unique ways dependent on the receptor type (i.e. affinity between ligand and GPCR). The potential negative regulators are *twk-16*, *frpr-4*, *flp-25*, *npr-4*, and *npr-9* (Supplementary Fig. 7a). The list of genes that regulate sleep, even in simple animals like *C. elegans*, continues to grow and suggests that throughout evolution, multiple and potentially redundant opposing mechanisms have arisen. As with timing, it will be important to determine on which cells these molecules function and the underlying mechanisms.

### Glutamate signaling and sleep

Glutamatergic transmission is conserved across phylogeny and both positively and negatively regulates circadian sleep in *Drosophila* and mammals (John et al. 2008; Zimmerman et al. 2008; Guo et al. 2016; Yoon et al. 2025). In *C. elegans*, glutamate signaling via the *glr-2* receptor promotes arousal during DTS (Choi et al. 2015), and here, we identified that glutamate signaling via *glr-5* positively regulates SIS. Interestingly, *glr-5* acts directly on the RIS sleep neuron, as well as AIB and RIM, which are gapped to RIS, to regulate sleep maintenance and timing. Numerous glutamatergic neurons are presynaptic to these cells; an important next step will be to identify how glutamate transmission communicates with sleep neurons (Supplementary Fig. 7b).

Some recent evidence supports a conserved function for glutamate signaling, considering the *Drosophila* ortholog Eye-enriched kainate receptor (Ekar) was identified in a genetic screen as a sleep-regulating gene (Stirtz et al. 2026). Also, the mammalian ortholog GRIK4 has been implicated with sleep regulation in mice. GRIK4-positive thalamic neurons are activated in response to arousal from slow-wave sleep (Shin et al. 2023). Thus, glutamate signaling through *glr-5*-like receptors may be a common mechanism of sleep coordination, maintenance, and arousal across phylogeny.

## Supplementary Material

iyag130_Supplementary_Data

## Data Availability

The data underlying this article are available in the article and in its online supplementary material. Additional details are available upon request to the corresponding author. All strains and reagents described in this manuscript are available upon request. Supplemental material available at GENETICS online.
