# Supplementary material for: A screen for stress-induced sleep genes in Caenorhabditis elegans reveals a role for glutamate signaling
Source: Genetics. 2026 May 20;233(4):iyag130. doi: 10.1093/genetics/iyag130 (PMC13439695; doi:10.1093/genetics/iyag130)
Supplement: Supplementary file 1 [file iyag130_supplementary_data.zip › Supplementary_Figure_Legends_GENETICS-2026-309132.docx]

**A screen for stress-induced sleep genes in *C. elegans* reveals a role for glutamate signaling**

Quinn Howe^1#^, Caroline Kominick^1#^, Mercedes Pierce^1^, Gina Gazarra^1^, Caroline E. Curtin^1^, Santino Diana^1^, Fredy Abboud^1^, Jordan Olenginski^1^, Mary Frattara^1^, Ting Brown^1^, Teagan McCarthy^1^, Paige Conrad^1^, Lauren Yoslov^1^, Rachel Vemula^1^, Anthony Gargani^1^, Emily Le^1^, and Matthew D. Nelson^1*^

^1^Saint Joseph’s University, Department of Biology, Philadelphia, PA 19131

^#^Equal contribution to this work

*Corresponding author

Email: [mnelson@sju.edu](mailto:mnelson@sju.edu) (MDN)

**Supporting Information**

**Figure S1: Overexpression of some neuropeptides can alter sleep. A)** Average number of body bends in one minute, two-hours post heat shock (30 minutes at 33°C), in wild-type (N=20), and transgenic strains over-expressing *flp-13* (N=19), *flp-3* (N=17), and *flp-33* (N=17) animals. The x-axis labels indicate the gene that is ectopically overexpressed (OE) by the heat-shock promoter. Statistical analyses were performed by one-way ANOVA followed by Tukey’s test (****p<0.0001). **B)** Quiescence in wild-type (N=60) and *nlp-1*(+) (N=64) animals. **C)** Quiescence in wild-type (N=91) and *nlp-61*(+) (N=94) animals. Right panel – *p<0.05 at 70-390, 410, 430, and 450-460 minutes. For **B** and **C**, sleep is defined as movement quiescence during UV-induced sleep. **D)** Schematic of the gene and allele structure, and peptide sequence for *nlp-61*. **E)** Schematic of the gene and allele structure for *frpr-11*, *frpr-12*, and *frpr-13*. For B and C, Student’s t-test was used to compare total quiescence (Left panels) and two-way ANOVA followed by Sidak’s test was used for temporal analyses (Right panels) (*p<0.05, ****p<0.0001).

**Figure S2: Developmentally-timed sleep of *nlp-1*, *nlp-61*, *twk-16*, and *glr-5* mutants.** For each panel, the average total minutes of movement quiescence during L4 DTS is displayed on the left, and the average minutes of movement quiescence in 10-minute windows during L4 DTS on the right. **A)** Quiescence in wild-type (N=42) and *nlp-1*(*ok1469*) (N=61) animals. **B)** Quiescence in wild-type (N=19) and *nlp-61*(*stj524*) (N=29) animals. **C)** Quiescence in wild-type (N=23) and *twk-16*(*stj13*) (N=24) animals. **D)** Quiescence in wild-type (N=21) and *glr-5*(*stj554*) (N=34) animals. Student’s t-test was used for comparison of total sleep between genotypes, and temporal comparisons were made using two-way ANOVA and Sidak’s test (***p<0.001). For each panel, sleep is defined as quiescence of movement.

**Figure S3: Other timing defects related to *nlp-1* and *npr-38*. A)** Quiescence in wild-type (N=58), *npr-38*(*stj367*) (N=60), and *npr-38*(*stj367*) transgenic (RIF rescue; Promoter: *nlp-6*) (N=110) animals. **B)** Quiescence in wild-type (N=92), *npr-38*(*stj367*) (N=66), and *npr-38*(*stj367*) transgenic (RIM rescue; Promoter: *cex-1*) (N=145) animals. **C)** Quiescence in wild-type (N=24), *nlp-61*(+) (N=24), and *npr-38*(*stj367*) transgenic (*nlp-61*(+)) (N=51) animals. **D)** Quiescence in wild-type (N=37), *npr-38*(+) (N=31), and *nlp-61*(*stj524*) transgenic (*npr-38*(+)) (N=70) animals. **E)** Quiescence in wild-type (N=33), *npr-38*(+) (N=26), and *nlp-1*(*ok1469*) transgenic (*npr-38*(+)) (N=42) animals. For **A-E**, statistical significance was calculated by one-way ANOVA followed by Tukey’s test (*p<0.05, **p<0.01, ***p<0.001, ****p<0.0001), and sleep is defined as quiescence of movement during UV-induced sleep. **F)** Average fraction of quiescent animals in the presence of noxious heat (37°C) in wild-type (6 trials, N=10 animals each) and *npr-38*(+) animals (7 trials, N=10 animals each). **G)** Average fraction of quiescent animals in the presence of noxious heat (37°C) in wild-type (8 trials, N=10 animals each) and *nlp-1*(*ok1469*) animals (10 trials, N=10 animals each). Significance was calculated by two-way ANOVA followed by Sidak’s multiple comparisons test (*p<0.05).

**Figure S4: Pumping rate of *eat-4* mutants. A)** Average pumps in 20 seconds in wild-type (N=48), *eat-4*(*ad450*) (N=56), *eat-4*(*nj6*) (N=51), and *eat-4*(*stj606*) (N=50) animals in the absence of auxin. **B)** Average pumps in 20 seconds in wild-type, *eat-4*(*stj606*), and transgenic *eat-4*(*stj606*) animals expressing *tir1* in all cells driven from the *eft-3* promoter (*eft-3p*::*tir1*), in the presence of auxin for the time-period displayed below the bars. One-way ANOVA followed by Tukey’s test was used to make comparisons between genotypes and conditions (***p<0.001).

**Figure S5: The response to acute stress in *glr-5*(*stj554*) mutants. A)** Average body bends performed in 1 minute following exposure to a 37°C heat stress for the designated number of minutes (min). **B)** Average fraction of quiescent animals in the presence of noxious heat (37°C) in wild-type (10 trials, N=10 animals each) and *glr-5*(*stj554*) animals (10 trials, N=10 animals each). Significance was calculated by two-way ANOVA followed by Sidak’s multiple comparisons test (*p<0.05). **C)** Average body bends performed in 1 minute (min) in the presence of blue light. For **A** and **C**, Student’s t-test was used to make comparisons between genotype (*p<0.05, **p<0.01,***p<0.001, ****p<0.0001). For **B**, significance was calculated by two-way ANOVA followed by Sidak’s multiple comparisons test (*p<0.05).

**Figure S6: *glr-5*(*tm3506*) mutants display enhanced sleep. A)** Quiescence in wild-type (N=52), *glr-5*(*stj554*) (N=77), and *glr-5*(*tm3506*) (N=87) animals. Statistical significance was calculated by one-way ANOVA followed by Tukey’s test (*p<0.05, ****p<0.0001). Sleep is defined as quiescence of movement during UV-induced sleep. **B)** Gene and protein structure for *glr-5* and the alleles used in this study.

**Figure S7: A proposed neuropeptidergic and glutamatergic model of sleep regulation. A)** A schematic of a proposed role for each gene during sleep initiation and/or maintenance. **B)** Glutamatergic neurons (*eat-4*-expressing) with direct synaptic connections to AIB, RIM, and RIS.
