# Supplementary material for: A screen for stress-induced sleep genes in Caenorhabditis elegans reveals a role for glutamate signaling
Source: Genetics. 2026 May 20;233(4):iyag130. doi: 10.1093/genetics/iyag130 (PMC13439695; doi:10.1093/genetics/iyag130)
Supplement: Supplementary file 1 [file iyag130_supplementary_data.zip › Table_S1_GENETICS-2026-309132.docx]

| **Strain Name** | **Genotype** | **Description** | **Location** |
| --- | --- | --- | --- |
| VC2497 | *flp-3*(*ok3265*) | 457bp deletion | CGC |
| NQ602 | *flp-13*(*tm2427*) | 380bp deletion | Raizen Lab |
| VC1982 | *flp-25*(*gk1016*) | 673bp deletion | CGC |
| VC2422 | *flp-33*(*gk1037*) | 507bp deletion | CGC |
| SJU322 | *flp-3*(*ok3265*);*flp-33*(*gk1037*) | VC2397 crossed with VC2422 | Nelson Lab |
| RB1340 | *nlp-1*(*ok1469*) | 700bp deletion | CGC |
| RB1341 | *nlp-1*(*ok1470*) | 600bp deletion | CGC |
| VC2357 | *nlp-38*(*ok2330*) | 1000bp deletion | CGC |
| TM1782 | *npr-4*(*tm1782*) | 1226bp deletion | NBRP |
| VC4220 | *frpr-16*(*gk5305*[*loxP*;*myo-2p*::*gfp*::*unc-54* 3' UTR;  *rps-27p*::*neoR*::*unc-54* 3' UTR; *loxP*]) | 1685bp deletion | CGC |
| TM1652 | *npr-9*(*tm1652*) | 318 bp deletion + 2 bp insertion | NBRP |
| RB1365 | *npr-16*(*ok1541*) | 145bp deletion | CGC |
| CHS1058 | *npr-12*(*yum1406*)*; npr-9*(*yum1404*);*npr-11*(*yum1405*) | Frameshift deletion and insertion of stop codon (Pu *et al.* 2023) | CGC |
| IK602 | *eat-4*(*nj6*) | Substitution – loss of function | CGC |
| DA572 | *eat-4*(*ad572*) | Substitution – loss of function | CGC |
| TM3506 | *glr-5*(*tm3506*) | 245bp deletion | NBRP |
| NQ385 | *frpr-4*(*ok2376*) | 1540bp deletion | Raizen Lab |
| NQ769 | *frpr-6*(*tm6135*) | 889 bp deletion | Nelson Lab |
| NQ915 | *dmsr-1*(*qn45*) | 278bp indel | Raizen Lab |
| SJU210 | *twk-16*(*stj13*) | *7*bp insertion in exon 4 – tgctagc | Nelson Lab |
| SJU253 | *frpr-4*(*stj21*) | 6bp insertion | Nelson Lab |
| SJU261 | *twk-16*(*stj16*) | *7*bp insertion in exon 6 – tgctagc | Nelson Lab |
| SJU269 | *twk-16*(*stj13*);stjEx181[*twk-16*(+);*myo-2p*::*mCherry*] | Rescue of *twk-16* with a genomic PCR fragment spanning ~8kb (5’UTR, coding, and 3’UTR) | Nelson Lab |
| SJU277 | *frpr-11*(*stj22*);*frpr-12*(*stj23*) | *frpr-11* – 6bp insertion in exon 4 – gctagc  *frpr-12 – 7*bp insertion in exon 4 – agctagc | Nelson Lab |
| SJU285 | *stjEx185*[*hsp-16.2p*::*flp-3*;*myo-2p*::*mCherry*] | *flp-3* overexpression | Nelson Lab |
| SJU287 | *stjEx187*[*hsp-16.2p*::*flp-33*;*myo-2p*::*mCherry*] | *flp-33* overexpression | Nelson Lab |
| SJU361 | *stjEx227*[*npr-38*(+);*myo-2p*::*gfp*] | *npr-38* overexpression |  |
| SJU471 | *frpr-11*(*stj22*);*frpr-12*(*stj23*);*frpr-13*(*stj458*) | *frpr-13* – 9bp insertion in exon 3 – gctagctag | Nelson Lab |
| SJU524 | *nlp-61*(*stj524*) | 12bp insertion in exon 1 – taagctagctaa | Nelson Lab |
| SJU554 | *glr-5*(*stj554*) | 13bp insertion in exon 3 – taattaggctagc | Nelson Lab |
| SJU561 | *glr-5*(*stj554*);*stj351*[*flp-21p*::*glr-5*; *myo-2*::*mCherry*] | RMG rescue – *glr-5* | Nelson Lab |
| SJU562 | *glr-5*(*stj554*);*stj352*[*flp-21p*::*glr-5*; *myo-2*::*mCherry*] | RMG rescue – *glr-5* | Nelson Lab |
| SJU573 | *stjEx362*[*nlp-61*(+);*myo-2p*::*mCherry*] | *nlp-61* overexpression | Nelson Lab |
| SJU578 | *glr-5*(*stj554*);*stjEx366*[*ver-3p*::*glr-5*; *flp-11p*::*glr-5*; *myo-2p*::*mCherry*] | ALA and RIS rescue – *glr-5* | Nelson Lab |
| SJU581 | *glr-5*(*stj554*);*stjEx369*[*glr-5*(+); *myo-2p*::*mCherry*] | Genomic rescue – *glr-5* | Nelson Lab |
| SJU594 | *glr-5*(*stj554*);*stjEx379*[*npr-9p*::*glr-5*;*myo-2p*::*mCherry*] | AIB rescue – *glr-5* | Nelson Lab |
| SJU595 | *glr-5*(*stj554*);*stjEx380*[*npr-9p*::*glr-5*;*myo-2p*::*mCherry*] | AIB rescue – *glr-5* | Nelson Lab |
| SJU606 | *eat-4*(*stj606*) | Insertion of degron sequence (Zhang *et al.* 2015) in place of stop codon at 3’ end | Nelson Lab |
| SJU611 | *nlp-61*(*stj524*);*stjEx227*[*npr-38*(+);*myo-2p*::*gfp*] | *nlp-61 lof* and *npr-38* overexpression | Nelson Lab |
| SJU614 | *nlp-1*(*ok1469*);*stjEx227*[*npr-38*(+);*myo-2p*::*gfp*] | *nlp-1 lof* and *npr-38* overexpression | Nelson Lab |
| SJU627 | *stjEx404*[*nlp-1*(+); *myo-2p*::*mCherry*] | *nlp-1* overexpression | Nelson Lab |
| SJU638 | *npr-38*(*stj367*);*stjEx412*[*cex-1p*::*npr-38*;*myo-2p*::*mCherry*] | RIM rescue – *npr-38* | Nelson Lab |
| SJU639 | *npr-38*(*stj367*);*stjEx413*[*cex-1p*::*npr-38*;*myo-2p*::*mCherry*] | RIM rescue – *npr-38* | Nelson Lab |
| SJU641 | *glr-5*(*stj554*);*stjEx415*[*twk-16p*::*glr-5*;*myo2p*::*mCherry*] | DVA rescue – *glr-5* | Nelson Lab |
| SJU644 | *glr-5*(*stj554*);*stjEx418*[*cex-1p*::*glr-5*;*myo-2p*::*mCherry*] | RIM rescue – *glr-5* | Nelson Lab |
| SJU651 | *npr-38*(*stj367*);*stjEx425*[nlp-6*p*::*npr-38*;*myo-2p*::*mCherry*] | RIF rescue – *npr-38* | Nelson Lab |
| SJU660 | *glr-5*(*stj554*);*stjEx429*[aptf-1*p*::*glr-5*;*cex-1p*::*glr-5*;*myo-2p*::*mCherry*] | AIB, RIS, and RIM rescue – *glr-5* | Nelson Lab |

**Table S1: Strains used in the current study.**
