# Supplementary material for: A screen for stress-induced sleep genes in Caenorhabditis elegans reveals a role for glutamate signaling
Source: Genetics. 2026 May 20;233(4):iyag130. doi: 10.1093/genetics/iyag130 (PMC13439695; doi:10.1093/genetics/iyag130)
Supplement: Supplementary file 1 [file iyag130_supplementary_data.zip › Table_S2_GENETICS-2026-309132.docx]

| **Genotype** | **Sleep Timing** | **Sleep Maintenance** |
| --- | --- | --- |
| *flp-3*(*ok3265*) | No change | No change |
| *flp-13*(*tm2427*) | Late | Reduced |
| *flp-25*(*gk1016*) | No change | Enhanced |
| *flp-33*(*gk1037*) | No change | No change |
| *flp-3*(*ok3265*);*flp-33*(*gk1037*) | Late | No change |
| *nlp-1*(*ok1469*) | Early | No change |
| *nlp-1*(*ok1470*) | Early | No change |
| *nlp-38*(*ok2330*) | No change | Reduced |
| *nlp-61*(*stj524*) | No change | Reduced |
| *npr-4*(*tm1782*) | No change | Enhanced |
| *frpr-16*(*gk5305*) | No change | No change |
| *npr-9*(*tm1652*) | No change | Enhanced |
| *npr-16*(*ok1541*) | No change | No change |
| *npr-12*(*yum1406*)*; npr-9*(*yum1404*);*npr-11*(*yum1405*) | No change | Enhanced |
| *frpr-4*(*ok2376*) | No change | Enhanced |
| *frpr-4*(*stj21*) | No change | Enhanced |
| *frpr-6*(*tm6135*) | No change | No change |
| *dmsr-1*(*qn45*) | Late | Reduced |
| *frpr-11*(*stj22*);*frpr-12*(*stj23*) | No change | Reduced |
| *frpr-11*(*stj22*);*frpr-12*(*stj23*);*frpr-13*(*stj458*) | No change | Reduced |
| *twk-16*(*stj13*) | No change | Enhanced |
| *twk-16*(*stj16*) | No change | Enhanced |
| *eat-4*(*nj6*) | No change | Reduced |
| *eat-4*(*ad572*) | No change | Reduced |
| *glr-5*(*tm3506*) | No change | Enhanced |
| *glr-5*(*stj554*) | Late | Reduced |

**Table S2: Sleep phenotypes of mutants in this study.**
