# Supplementary material for: A screen for stress-induced sleep genes in Caenorhabditis elegans reveals a role for glutamate signaling
Source: Genetics. 2026 May 20;233(4):iyag130. doi: 10.1093/genetics/iyag130 (PMC13439695; doi:10.1093/genetics/iyag130)
Supplement: Supplementary file 1 [file iyag130_supplementary_data.zip › Table_S3_GENETICS-2026-309132.docx]

| **Oligo Name** | **Sequence** | **Description** | **Application** |
| --- | --- | --- | --- |
| oSJUcrDNA35 | TCTCTATTTATTTTTTAATTTTCCAGGTCCAAATCAgctagcTGGTGTCGATACACCACATCATCATACTCCATCGC | Repair template for *frpr-4*(*stj21*) | Insertion mutant  CRISPR  *frpr-4*(*stj21*) |
| oSJUcrDNA36 | CATTGAGAAGAAGTGGCAGAATC | Forward primer for *stj21* screening  Pair – oSJUcrDNA37 |  |
| oSJUcrDNA37 | GATTGCTGTTTGTCGACCACTTG | Reverse primer for *stj21* screening  Pair – oSJUcrDNA36 |  |
| oSJUcrRNA30 | TGGTGTATCGACACCAGATTGTTTTAGACTATGC | gRNA for *frpr-4* |  |
| oSJUcrDNA139 | TCTCCTCTCGTACATTTTCTACAATGAGCCACTGGtaagctagctaaATTGCAACTCTTCTTGCCTTCATCCTCATTGCCAA | Repair template for *nlp-61*(*stj524*) | Insertion mutant  CRISPR  *nlp-61*(*stj524*) |
| oSJUcrDNA140 | GTTGAAAGCTCAGAAATTTCTATTAG | Forward primer for *stj524* screening  Pair – oSJUcrDNA141 |  |
| oSJUcrDNA141 | CTGGAAATAACACTAATTGCGAC | Reverse primer for *stj524* screening  Pair – oSJUcrDNA140 |  |
| oSJUcrDNA26 | CACTAAAAATTGTGTGAACGGAACCAATTTCATGGgctagcTAACCGGGGCTGAAAATGAATTTTGAATATTTGTT | Repair template for *frpr-11*(*stj22*) | Insertion mutant  CRISPR  *frpr-11*(*stj22*) |
| oSJUcrDNA29 | GAACAAAAACACGGTAAGCAATG | Forward primer for *stj22* screening  Pair – oSJUcrDNA30 |  |
| oSJUcrDNA30 | GACTTTTTAAAAGACGCCTTG | Reverse primer for *stj22* screening  Pair – oSJUcrDNA29 |  |
| oSJUcrRNA12 | GGAACCAATTTCATGGTAACCGG | gRNA for *frpr-11* |  |
| oSJUcrDNA27 | ATTTTCAGTGCATTTTGTTTAGTATACATTTATCCagctagcATTCGGGTGCATGGCAAAAATGATTGGTCAGTACA | Repair template for *frpr-12*(*stj23*) | Insertion mutant  CRISPR  *frpr-12*(*stj23*) |
| oSJUcrDNA31 | TTTTCCCGGTGTTCTTTTTTG | Forward primer for *stj23* screening  Pair – oSJUcrDNA32 |  |
| oSJUcrDNA32 | CACAAACTACTCTTTCCTTCAATAAG | Reverse primer for *stj23* screening  Pair – oSJUcrDNA31 |  |
| oSJUcrRNA13 | TAGTATACATTTATCCATTCGGG | gRNA for *frpr-12* |  |
| oSJUcrDNA42 | ACAGCACTTGGCGCTCGCTACAATTCCTCTTTTCAgctagctagTCGTTGGCCTAATTTTGACAGGAATATCAATTTAT | Repair template for *frpr-13*(*stj458*) | Insertion mutant  CRISPR  *frpr-13*(*stj458*) |
| oSJUcrDNA43 | GACAGCTCATTTCTGATGAAC | Forward primer for *stj458* screening  Pair – oSJUcrDNA44 |  |
| oSJUcrDNA44 | CATTTTCAGTTGTTGGGCATG | Reverse primer for *stj458* screening  Pair – oSJUcrDNA43 |  |
| oSJUcrRNA30 | ACAATTCCTCTTTTCATCGTTGG | gRNA for *frpr-13* |  |
| oSJUcrDNA10 | AAGCGAATGGAATTTTGTAACTGCAACGTTATATGtgctagcGTTTTGGCATTGTCACAACATTAGGTACATCATGA | Repair template for *twk-16*(*stj13*) | Insertion mutant  CRISPR  *twk-16*(*stj13*) |
| oSJUcrDNA12 | GTTAATTGGTTTGATGAGAATTCC | Forward primer for *stj13* screening  Pair – oSJUcrDNA13 |  |
| oSJUcrDNA13 | CTGCAAAATTGTTCAGGTACTG | Reverse primer for *stj13* screening  Pair – oSJUcrDNA12 |  |
| oSJUcrRNA5 | ACTGCAACGTTATATGGTTTTGG | gRNA for *twk-16*(*stj13*) |  |
| oSJUcrDNA11 | CCGGCAAAATTTACAAAGAATCTTCAATCCAAGTCtgctagcACATCGTTAGCTCTTCTATGTGTCTTCTTAATATA | Repair template for *twk-16*(*stj16*) | Insertion mutant  CRISPR  *twk-16*(*stj16*) |
| oSJUcrDNA14 | CAGTACCTGAACAATTTTGCAG | Forward primer for *stj13* screening  Pair – oSJUcrDNA15 |  |
| oSJUcrDNA15 | CTAACTGATAGCAATTGTTGTAATC | Reverse primer for *stj13* screening  Pair – oSJUcrDNA14 |  |
| oSJUcrRNA6 | GAAGAGCTAACGATGTGACTTGG | gRNA for *twk-16*(*stj16*) |  |
| oSJUcrDNA155 | CTTGCATCTCTTCAAATGGGACTCATTGAGTTGAAtaattaggctagcGCACTGGTTTCTGCTCACCAATATGGAGCTTTCCA | Repair template for *glr-5*(*stj554*) | Insertion mutant  CRISPR  *glr-5*(*stj554*) |
| oSJUcrDNA156 | CTGGAACAACTCTTGGAAAGTG | Forward primer for *stj554* screening  Pair – oSJUcrDNA157 |  |
| oSJUcrDNA157 | GACAACTCGTTGAACACATTGG | Reverse primer for *stj554* screening  Pair – oSJUcrDNA156 |  |
| oSJUcrRNA29 | GACTCATTGAGTTGAAGCACTGG | gRNA for *glr-5*(*stj554*) |  |
| oSJUcrDNA178 | ATGAGCTCATAAAATTTGAAAATTTACAGCTCCTGGAACCAACCCGTTCGCTTCAGCTTGGGATGAACATGGATCCTCTGGAGTTGTGGAAAATCCGCATatgcctaaagatccagccaaa | Forward primer (long homology arms) – degron sequence for *eat-4*  Pair – oSJUcrDNA179 | Insertion mutant  CRISPR  *eat-4*(*stj606*) |
| oSJUcrDNA179 | AACATAAGATATTAGGAATAACTGAGAGAATTAAATAAATAAATTAAAATTTTTAGAAGGTATCCTAACTAGACGTTATTTATCACTACCACTGCTGATActtcacgaacgccgccgcctc | Reverse primer (long homology arms) – degron sequence for *eat-4*  Pair – oSJUcrDNA178 |  |
| oSJUcrDNA180 | AACATGGATCCTCTGGAGTTGTGGAAAATCCGCATatgcctaaagatccagccaaa | Forward primer (short homology arms) – degron sequence for *eat-4*  Pair – oSJUcrDNA181 |  |
| oSJUcrDNA181 | TAACTAGACGTTATTTATCACTACCACTGCTGATActtcacgaacgccgccgcctc | Reverse primer (short homology arms) – degron sequence for *eat-4*  Pair – oSJUcrDNA180 |  |
| oSJUcrRNA32 | CCGCATTATCAGCAGTGGTAGTGA | gRNA for *glr-5*(*stj606*) |  |
| oSJU18 | GAAGTTTTTTAGATGCACTAG | 5’ of *hsp-16.2*  Pair – oSJU577 | Ectopic over-expression  *flp-3* |
| oSJU19 | GTTTTTTAGATGCACTAGAAC | Nested to oSJU18  Pair – oSJU580 or oSJU584 |  |
| oSJU577 | ATGAGGTGATTGGGCGATATCATGATTATAGTTTGAAGATTTCTAATTTC | -1 to *hsp-16.2*, adds *flp-3* tail  Pair – oSJU18 |  |
| oSJU578 | TGTGAAATTAGAAATCTTCAAACTATAATCATGATATCGCCCAATCACCTC | +1 to *flp-3*, adds *hsp-16.2* tail  Pair – oSJU579 |  |
| oSJU579 | GTTTCCACGCATTCATTGGTTG | 3’ of *flp-3*  Pair – oSJU578 |  |
| oSJU580 | CATTGGTTGGCACATGTTGGTC | Nested to oSJU579  Pair – oSJU19 |  |
| oSJU581 | ACGATGAGAATAAGAAAACGCATGATTATAGTTTGAAGATTTCTAATTTC | -1 to *hsp-16.2*, adds *flp-33* tail  Pair – oSJU18 | Ectopic over-expression  *flp-33* |
| oSJU582 | TGAAATTAGAAATCTTCAAACTATAATCATGCGTTTTCTTATTCTCATCGTC | +1 to *flp-33*, adds *hsp-16.2* tail  Pair – oSJU583 |  |
| oSJU583 | GTATTTTGATGCCCGGTGACATG | 3’ of *flp-33*  Pair – oSJU582 |  |
| oSJU584 | GTGACATGTCAATACGCACTTC | Nested to oSJU583  Pair – oSJU19 |  |
| oSJU1335 | CTTTCGTACCAGAATTTCACTA | 5’ of *nlp-1*  Pair – oSJU1336 | Multi-copy overexpression  *nlp-1* |
| oSJU1336 | TAATGTTTCACATTCCAGTCG | 3’ of *nlp-1*  Pair – oSJU1335 |  |
| oSJU1017 | CTGGCATGTAGTGCGTAAATC | Forward - 5’ of *nlp-61*  Pair – oSJU1018 | Multi-copy overexpression  *nlp-61* |
| oSJU1018 | CTTCCGTGTCATACAAGTGTAC | Reverse - 3’ of *nlp-61*  Pair – oSJU1017 |  |
| oSJU52 | CTTTGTAAGCTATGACGTCACTTC | Forward - 5’ of *twk-16*  Pair – oSJU54 | Genomic fragment for rescue  *twk-16* |
| oSJU54 | CATGCGCAGTAAACTTTATTC | Reverse - 3’ of *twk-16*  Pair – oSJU52 |  |
| oSJU1367 | CTGATCGTTGTATTTCTACTTGT | 5’ of *nlp-6p*  Pair – oSJU1377 | *npr-38* rescue in the RIF – *nlp-6* promoter |
| oSJU1368 | CTACTTGTTCACTTTCTTCTTTC | Nested to oSJU1367  Pair – oSJU1376 |  |
| oSJU1377 | ATTCTTTGTATGTCATCGCCTCCCATTGTACCTAAACAAAACTCATACAT | +3 to *nlp-6* and adds a *npr-38* tail  Pair – oSJU1367 |  |
| oSJU1378 | AAAATGTATGAGTTTTGTTTAGGTACAATGGGAGGCGATGACATACAAAG | +1 to *npr-38* and adds a *nlp-6* tail  Pair – oSJU1375 |  |
| oSJU1375 | TGCTGAATCCCAAGATAATCTG | 3’ of *npr-38*  Pair – oSJU1378 |  |
| oSJU1376 | CCAAGATAATCTGAAAGCTCT | Nested to 1375  Pair – oSJU1368 |  |
| oSJU1388 | GAGCTTCTTCTATAATGCCGAG | 5’ of *cex-1*  Pair – oSJU1394 | *npr-38* rescue in the RIM – *cex-1* promoter |
| oSJU1389 | CTTCTATAATGCCGAGTTTGTAAG | Nested to oSJU1388  Pair – oSJU1376 |  |
| oSJU1394 | TTCTTTGTATGTCATCGCCTCCCATTTCAGCGGCTTTTCGTAAGCT | +3 to *cex-1* and adds a *npr-38* tail  Pair – oSJU1388 |  |
| oSJU1395 | ACTGGAAGCTTACGAAAAGCCGCTGAAATGGGAGGCGATGACATACAAAG | +1 to *npr-38* and adds a *cex-1* tail  Pair – oSJU1375 |  |
| oSJU1166 | CAGCAGGTTTCACAAAGTTCA | 5’ to *glr-5*  Pair – oSJU1229 | Genomic fragment for rescue of *glr-5* |
| oSJU1229 | GATATGAACAAATTCTGATGTCAC | 3’ to *glr-5*  Pair – oSJU1229 |  |
| oSJU739 | CTAGTTTTGCATTTTCGCATTG | 5’ to *ver-3*  Pair – oSJU1134 | *glr-5* rescue in ALA – *ver-3* promoter |
| oSJU740 | GCATTTTCGCATTGTATTGATCTC | Nested to oSJU739  Pair – oSJU1137 |  |
| oSJU1134 | AAAATGTAGTTGTTATTAAACTGCATCTCAGAAAAATTGTTCTTGTGCAC | +3 to *ver-3* and adds a *glr-5* tail  Pair – oSJU739 |  |
| oSJU1135 | TCAGTGCACAAGAACAATTTTTCTGAGATGCAGTTTAATAACAACTACAT | +1 to *glr-5* and adds a *ver-3* tail  Pair – oSJU1136 |  |
| oSJU1136 | CTGCGAGAACTTTAGAGTAACAG | 3’ of *glr-5*  Pair – oSJU1135 |  |
| oSJU1137 | GTAACAGATATGAACAAATTCTG | Nested to 1136  Pair – oSJU740 |  |
| oSJU718 | CATTTTATCCTCATGCAGATGAAG | 5’ of *flp-11*  Pair – oSJU1146 | *glr-5* rescue in RIS – *flp-11* promoter |
| oSJU719 | CTCATGCAGATGAAGTCTTTC | Nested to oSJU718  Pair – oSJU1137 |  |
| oSJU1146 | AAAATGTAGTTGTTATTAAACTGCATTATTCAGTATGAACTGCAAAAAGT | +3 to *flp-11* and adds a *glr-5* tail  Pair – oSJU718 |  |
| oSJU1147 | TCACTTTTTGCAGTTCATACTGAATAATGCAGTTTAATAACAACTACAT | +1 to *glr-5* and adds a *flp-11* tail  Pair – oSJU1136 |  |
| oSJU1118 | GTGTTTTCGAACATTTTTCATG | 5’ of *flp-21*  Pair – oSJU1144 | *glr-5* rescue in RMG – *flp-21* promoter |
| oSJU1119 | GTGAGGGAAATTTCATAGACCA | Nested to oSJU1118  Pair – oSJU1137 |  |
| oSJU1144 | AGAAAATGTAGTTGTTATTAAACTGCATCGTCTGAAAATGACTTTTTGGA | +3 to *flp-21* and adds a *glr-5* tail  Pair – oSJU1118 |  |
| oSJU1145 | AAAATCCAAAAAGTCATTTTCAGACGATGCAGTTTAATAACAACTACAT | +1 to *glr-5* and adds a *flp-21* tail  Pair – oSJU1136 |  |
| oSJU912 | CATCGCCAGGCAAGCTATTAC | 5’ of *npr-9*  Pair – oSJU1142 | *glr-5* rescue in AIB – *npr-9* promoter |
| oSJU913 | GTAGCAGAAATCCAAATCCAAAC | Nested to oSJU912  Pair – oSJU1137 |  |
| oSJU1142 | GAAAATGTAGTTGTTATTAAACTGCATTTCCATTGTCAAAAAGTTGATGA | +3 to *npr-9* and adds a *glr-5* tail  Pair – oSJU912 |  |
| oSJU1143 | AAATCATCAACTTTTTGACAATGGAAATGCAGTTTAATAACAACTACAT | +1 to *glr-5* and adds a *npr-9* tail  Pair – oSJU1136 |  |
| oSJU1355 | CTTTGTAAGCTATGACGTCACTTC | 5’ of *twk-16*  Pair – oSJU1357 | *glr-5* rescue in DVA – *twk-16* promoter |
| oSJU1356 | GACGTCACTTCTTAACAAACGG | Nested to oSJU1355  Pair – oSJU1137 |  |
| oSJU1357 | AGAAAATGTAGTTGTTATTAAACTGCATTGTGGTATCTGGAAGATGGAT | +3 to *twk-16* and adds a *glr-5* tail  Pair – oSJU1355 |  |
| oSJU1358 | TTTTTGCAATCCATCTTCCAGATACCACAATGCAGTTTAATAACAACTAC | +1 to *glr-5* and adds a *twk-16* tail  Pair – oSJU1136 |  |
| oSJU1390 | GAGAAAATGTAGTTGTTATTAAACTGCATTTCAGCGGCTTTTCGTAAGCT | +3 to *cex-1* and adds a *glr-5* tail  Pair – oSJU1388 | *glr-5* rescue in RIM – *cex-1* promoter |
| oSJU1391 | ACTGGAAGCTTACGAAAAGCCGCTGAAATGCAGTTTAATAACAACTACAT | +1 to *glr-5* and adds a *npr-9* tail  Pair – oSJU1136 |  |

**Table S3: Oligonucleotides used in the current study (Integrated DNA Technologies).**
